# Supplementary material for: Whole-genome sequencing reveals progressive versus stable myeloma precursor conditions as two distinct entities
Source: Nat Commun. 2021 Mar 25;12:1861. doi: 10.1038/s41467-021-22140-0 (PMC7994386; doi:10.1038/s41467-021-22140-0)
Supplement: Supplementary file 1 — Supplementary Information [file 41467_2021_22140_MOESM1_ESM.pdf]

Whole genome sequencing reveals progressive versus stable myeloma precursor conditions as two distinct entities

Oben et al.

Supplementary Figures

Supplementary Fig. 1. Mutational burden across the three main clinical stages: multiple myeloma (brown), stable and progressive myeloma precursor condition (blue and purple, respectively). p values were calculated using Wilcoxon rank-sum test. Boxplots show the median and interquartile range. MM: multiple myeloma. MGUS: monoclonal gammopathy of undetermined significance; SMM: smoldering multiple myeloma; SD: stable disease; PD: progressive disease.

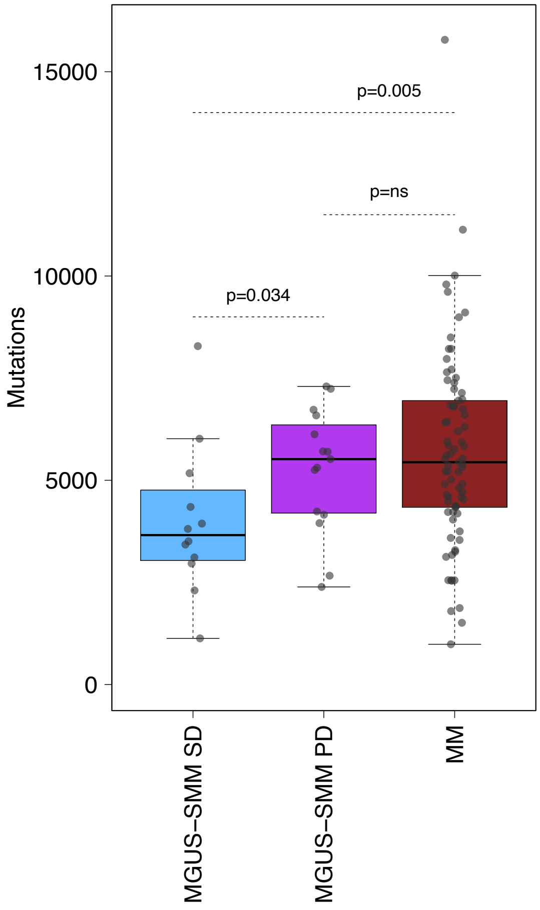

Supplementary Fig. 2. De novo extraction of mutational signatures. The eight mutational signatures extracted by sigprofiler.

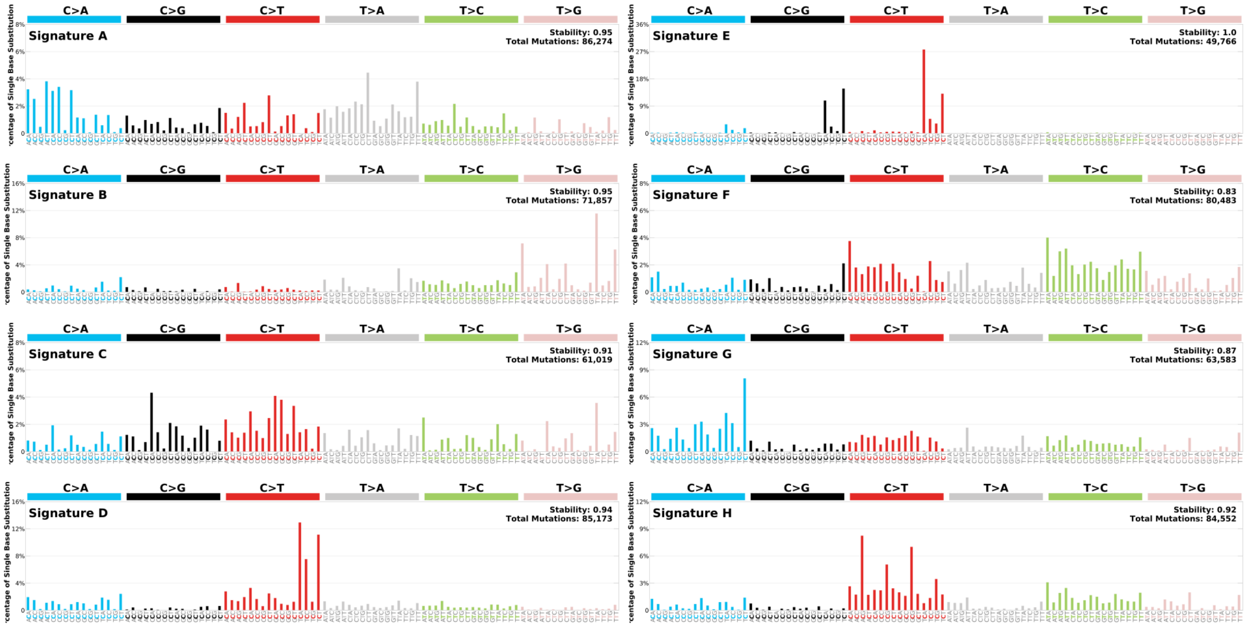

Supplementary Fig. 3. The prevalence of recurrent multiple myeloma aneuploidies across the three groups investigated in this study: multiple myeloma (brown bars), stable and progressive myeloma precursor condition (blue and purple bars, respectively). Stable myeloma precursor condition showed a significantly lower prevalence of all recurrent cytogenetic aberration but 1p36 DEL (see **Supplementary Table 4**). MM: multiple myeloma; MGUS: monoclonal gammopathy of undetermined significance; SMM: smoldering multiple myeloma; SD: stable disease; PD: progressive disease. DEL: deletion; AMP: gain.

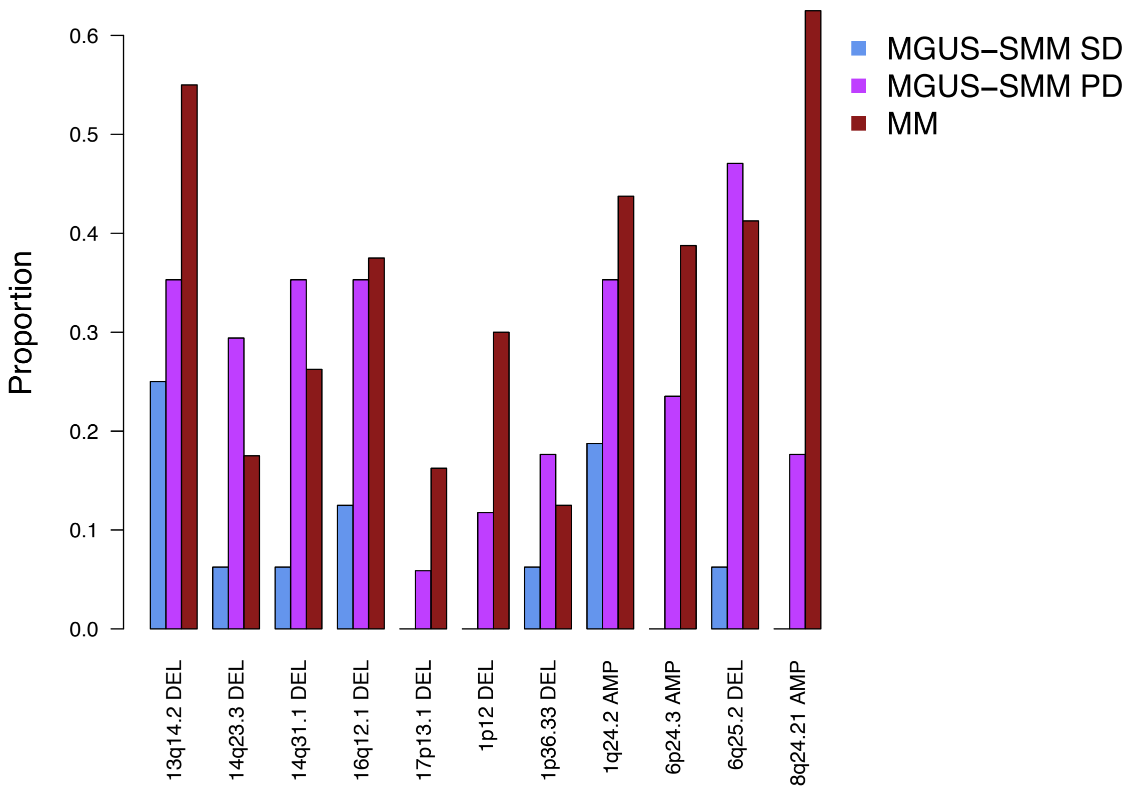

Supplementary Fig. 4. Two-dimensional density plot showing the phylogenetic tree of a patient with stable myeloma precursor condition for more than 15 years with one sample collected at diagnosis and one after 2 years of follow up. Increasing intensity of blue indicates higher mutation burden. The cancer cell fraction of each Dirichlet process cluster is reported with a red dot. The mutational burden of this patient's DP-clusters was: #1 = 2438; #2 = 1753; #3 = 711; 4# = 110. MGUS: monoclonal gammopathy of undetermined significance; SMM: smoldering multiple myeloma; SD: stable disease.

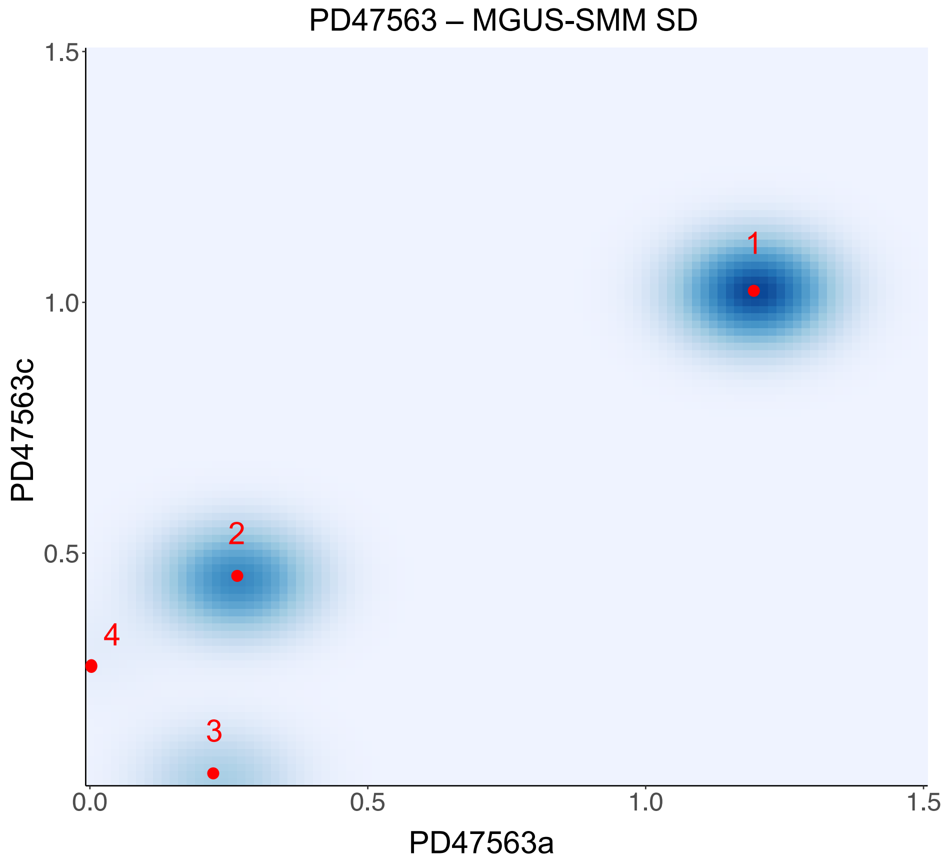

Supplementary Fig. 5. Structural variants breakpoint distribution across the genome. Asterisks reflect significant association between SV and distinct genomic features tested using linear regression model (*lm* R function). Events involving *IGH* locus were excluded due to their known strong association with super enhancer. Error bars reflect the 95% confidence interval generated by *lm* for each regression.

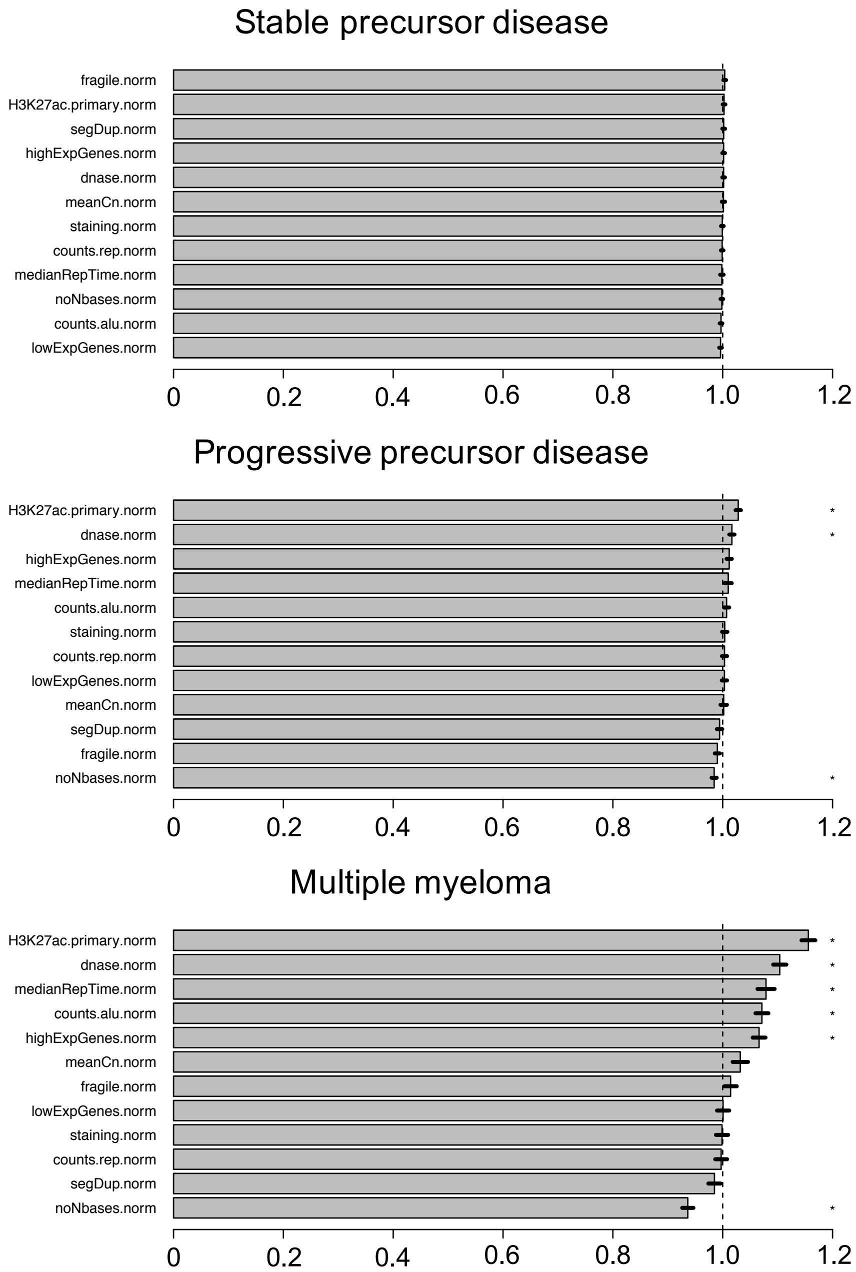

Supplementary Fig. 6. Prevalence of known structural variants (SV) hotspots (n=69) across the three clinical stages: multiple myeloma (brown), stable and progressive myeloma precursor condition (blue and purple, respectively). p values were calculated using Wilcoxon rank-sum test. Boxplots show the median and interquartile range. MM: multiple myeloma. MGUS: monoclonal gammopathy of undetermined significance; SMM: smoldering multiple myeloma; SD: stable disease; PD: progressive disease.

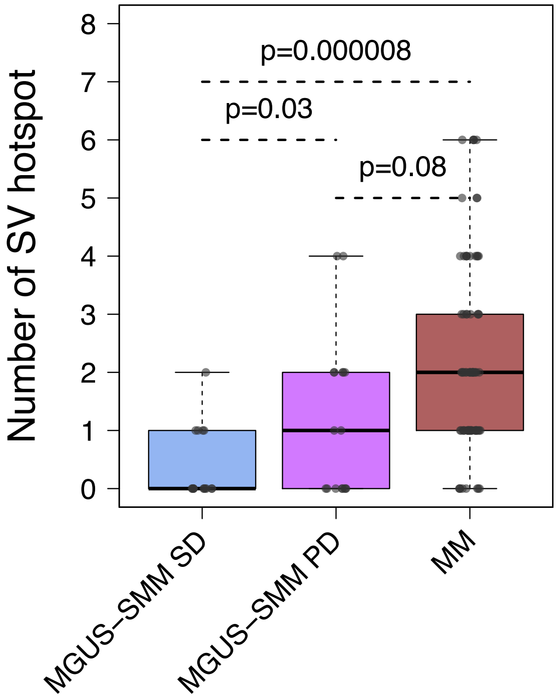

Supplementary Fig. 7. Coverage (a) and cancer cell fraction (b) of all cases included in this study. Boxplots show the median and interquartile range. MM: multiple myeloma. MGUS: monoclonal gammopathy of undetermined significance; SMM: smoldering multiple myeloma; SD: stable disease; PD: progressive disease. WGS: whole genome sequencing.

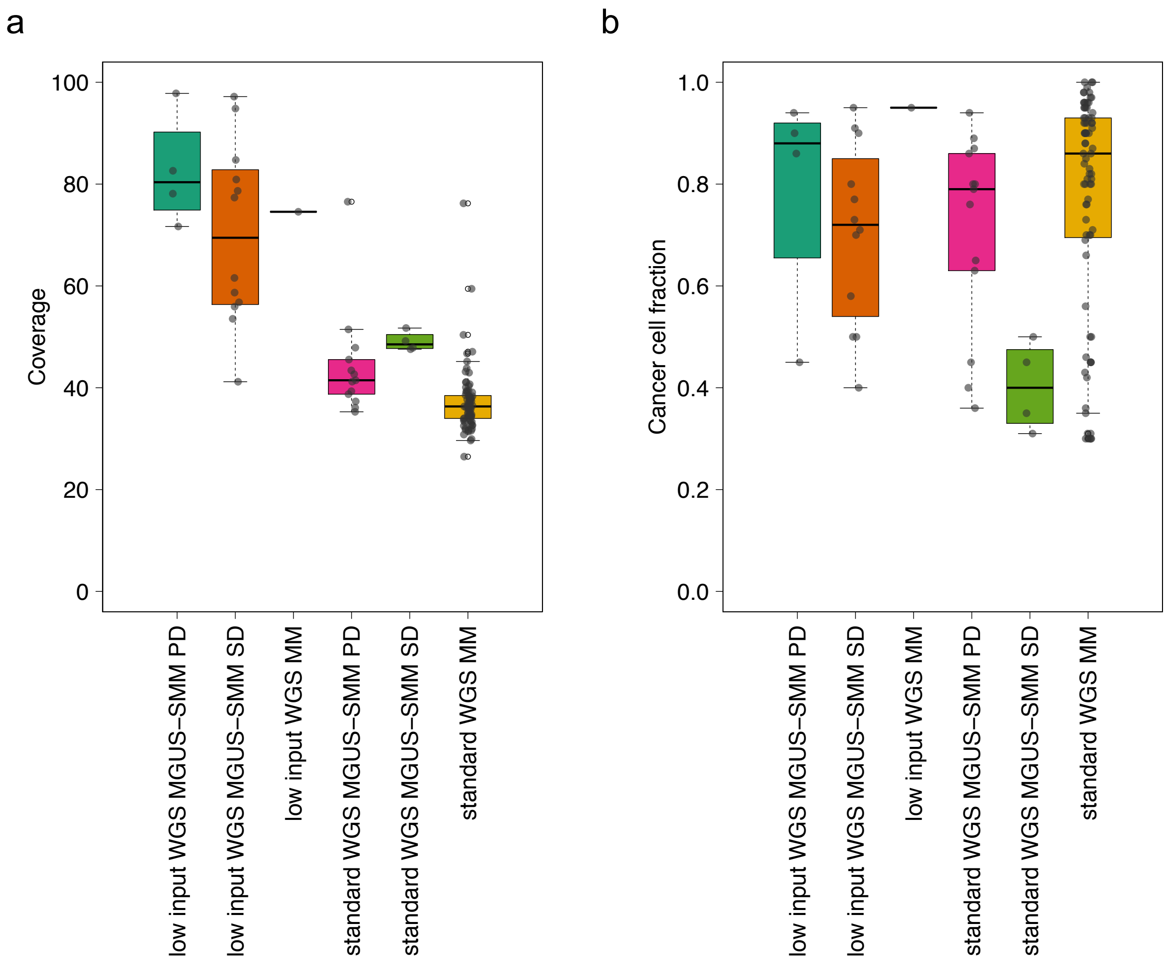

Supplementary Tables

Supplementary Table 1. Summary of the total number of selected CD138+ bone marrow plasmacell (BMPC) sequenced using low input WGS approach.

Sample	Amount BMPCs	Amount cells matched control
**PD47575a**	3000	6000
**PD47582a**	1518	2015
**PD47561a**	2285	6000
**PD47577a**	1000	3000
**PD47578a**	3000	3000
**PD47579a**	3000	3000
**PD47563a**	3000	3000
**PD47563c**	1495	
**PD47573a**	3000	3000
**PD47581a**	3000	3000
**PD47574a**	3000	6000
**PD47576a**	3000	3000
**PD47567a**	3000	3000
**PD47570a**	3000	3000
**PD47572a**	3000	3000
**PD47580a**	3000	3000
**PD47580c**	3000	

Supplementary Table 2. Percentage of bone marrow plasma cell (BMPC) at diagnosis for all cases with myeloma precursor condition included in this study. PD47576a has 10% BMPC at the first bone marrow evaluation, but <10% in all the subsequent follow up evaluations. MM: multiple myeloma. MGUS: monoclonal gammopathy of undetermined significance; SMM: smoldering multiple myeloma. WGS: whole genome sequencing.

Sample	Stage	BMPC (%)
**IID_H135336**	SMM - progressed	10
**IID_H196059**	MGUS - stable	7
**IID_H196061**	MGUS- stable	4
**IID_H196062**	SMM - stable	15
**IID_H196063**	MGUS- stable	5
**IID_H196064**	MGUS - progressed	8
**PD26400a**	SMM - progressed	33
**PD26401a**	SMM - progressed	21
**PD26402a**	SMM - progressed	40
**PD26403a**	SMM - progressed	29
**PD26404a**	SMM - progressed	37
**PD26405a**	SMM - progressed	25
**PD26406a**	SMM - progressed	NA
**PD26407a**	SMM - progressed	39
**PD26408a**	SMM - progressed	31
**PD26409a**	SMM - progressed	10
**PD26424a**	SMM - progressed	61*
**PD47561a**	MGUS- stable	3
**PD47563a**	MGUS- stable	3
**PD47567a**	MGUS - progressed	6.5
**PD47570a**	MGUS - progressed	5
**PD47572a**	MGUS - progressed	4.4
**PD47573a**	MGUS- stable	9.1
**PD47574a**	MGUS- stable	7
**PD47575a**	MGUS- stable	6
**PD47576a**	MGUS- stable	10
**PD47577a**	MGUS- stable	3.8
**PD47578a**	MGUS- stable	8.2
**PD47579a**	MGUS- stable	6
**PD47580a**	SMM - progressed	11.8
**PD47581a**	MGUS- stable	0.6
**PD47582a**	MGUS- stable	3.2

*this case was diagnosed in 2010 prior to the 2014 IMWG diagnostic criteria for MM. Patients was monitored for high risk smoldering multiple myeloma with a less than 60% clonal plasmacells.

Supplementary Table 3. Assignment of single base substitution (SBS) signatures extracted by *SigProfiler*. NMF: non-matrix factorization

De novo extracted	Global NMF Signatures	Similarity
**Signature 96-A**	Signature SBS8	0.93
**Signature 96-B**	Signature SBS9	0.93
**Signature 96-C**	Signature SBS-MM1	1
**Signature 96-D**	Signature SBS2	0.97
**Signature 96-E**	Signature SBS2 (56.80%) & Signature SBS13 (43.20%)	1
**Signature 96-F**	Signature SBS5	0.93
**Signature 96-G**	Signature SBS18	0.95
**Signature 96-H**	Signature SBS1 (23.14%) & Signature SBS5 (76.86%)	0.97

Supplementary Table 4. Different prevalence of recurrent multiple myeloma (MM) copy number variants (CNV) across the three study groups: MM, stable and progressive precursor conditions. Fisher's test was used to estimate the p value. MGUS: monoclonal gammopathy of undetermined significance; SMM: smoldering multiple myeloma; SD: stable disease; PD: progressive disease; DEL: deletion; AMP: amplification.

CNV event	p value MGUS/SMM SD vs MGUS/SMM PD	p value MGUS/SMM PD vs MM	p value MGUS/SMM SD vs MM
**13q14.2 DEL**	0.3908	0.1343	0.000002
**14q23.3 DEL**	0.0122	0.2063	0.0055
**14q31.1 DEL**	0.0028	0.4047	0.00002
**16q12.1 DEL**	0.0315	1	0.00001
**17p13.1 DEL**	0.1531	0.4849	0.000004
**1p12 DEL**	0.0222	0.1642	0.4e-11
**1p36.33 DEL**	0.1395	0.4637	0.13803
**1q24.2 AMP**	0.1965	0.616	0.00008
**6p24.3 AMP**	0.0003	0.3016	0.2e-15
**6q25.2 DEL**	0.0001	0.6218	0.4e-9
**8q24.21 AMP**	0.003	0.0004	0.2e-31

Supplementary Table 5. Summary of the clinical features of all cases with multiple myeloma with available SNP array data. ISS; International Staging System, IQR; inter-quartile range, NA; not available, OS; overall survival, PFS; progression-free survival

Variable	Value
**Patients (n)**	148
**Age at diagnosis (years, median, IQR)**	60.9 (54.3 - 69.1)
**Gender (female, male)**	56, 92
**ISS stage 1 (n, %)**	30 (20.3%)
**2 (n, %)**	60 (40.5%)
**3 (n, %)**	30 (20.3%)
**NA (n, %)**	28 (18.9%)
**Untreated (n, %)**	87 (58.8%)
**Post-treatment (n, %)**	61 (41.2%)
**PFS follow-up (years, median, IQR)**	2.2 (1.5 - 3.0)
**Deaths (n, %)**	20 (14%)
**OS follow-up (years, median, IQR)**	3.1 (2.2 - 5.1)

Supplementary Table 6. Summary of the clinical features of all cases with myeloma precursor condition with available SNP array data. HRCG; high risk cytogenetics (t(4;14), gain1q, del17p), FLC; free light chain, IQR; inter-quartile range, MGUS; monoclonal gammopathy of uncertain significance, SMM; smoldering multiple myeloma, PFS; progression-free survival

**Variable**	**Value**
**Patients (n)**	68
**Age (median, IQR)**	61.6 (53.6 - 67.9)
**Gender (female, male)**	34, 34
**MGUS (n, %)**	15 (22.1)
**SMM (n, %)**	53 (77.9)
**Plasma cells, >10% (n, %)**	45 (66.2)
**>20% (n, %)**	11 (16.2)
**M-spike, >2g/dL (n, %)**	7 (10.3)
**>3g/dL (n, %)**	2 (3.0)
**FLC ratio, >8 (n, %)**	26 (38.2)
**>20 (n, %)**	12 (17.6)
**HRCG (n, %)**	9 (13.2)
**Non-IgG isotype (n, %)**	25 (36.8)
**PFS follow-up (years, median, IQR)**	2.1 (1.1 - 4.0)
