# Supplementary material for: Whole-genome sequencing reveals progressive versus stable myeloma precursor conditions as two distinct entities
Source: Nat Commun. 2021 Mar 25;12:1861. doi: 10.1038/s41467-021-22140-0 (PMC7994386; doi:10.1038/s41467-021-22140-0)
Supplement: Supplementary file 3 — Description of Additional Supplementary Files [file 41467_2021_22140_MOESM3_ESM.pdf]

Description of Additional Supplementary Files

File Name: Supplementary Data 1
Description: Summary of the clinical and sequencing features of all samples included in this study

File Name: Supplementary Data 2
Description: Catalogue of 80 driver genes mutated in multiple myeloma. TSG= tumor suppressor gene

File Name: Supplementary Data 3
Description: Exploring pattern of positive selection in myeloma precursor disease
