# Supplementary material for: Whole-genome sequencing reveals progressive versus stable myeloma precursor conditions as two distinct entities
Source: Nat Commun. 2021 Mar 25;12:1861. doi: 10.1038/s41467-021-22140-0 (PMC7994386; doi:10.1038/s41467-021-22140-0)
Supplement: Supplementary file 6 — Supplementary Data 3 [file 41467_2021_22140_MOESM6_ESM.html]

Exploring pattern of positive selection in myeloma precursor disease


# Exploring pattern of positive selection in myeloma precursor disease

# Libraries

```
 library(dndscv)
```

# Load mutational data

```
 setwd("~/Desktop/project/MGUS_sanger/Fede_data/")
 compass = read.table("commpass_newly_diagnosed_mm.txt",sep="\t",header=T,stringsAsFactors=F)
 muts    = read.table("wgs_wxs_all_cases.txt",sep="\t",header=T,stringsAsFactors=F)
```

# Load catalogue of myeloma driver genes

```
### list if 81 drivers - remove IGLL5 and change CCND1 and MAF as AID target
 drivers = read.table("driver_fra_morgan.txt",sep="\t",header=T,stringsAsFactors=F)
 drivers$AID.target[drivers$Gene.Symbol %in% c("MAF","CCND1")]<-"yes"
 drivers$AID.target[drivers$AID.target==" "]<-""
 colnames(drivers)[1]<-"gene"
 drivers<- drivers[drivers$gene !="IGLL5",]
```

# Load clinical data

```
#### updated clinical file WGS
 all_final_clinic    = read.table("~/Desktop/project/MGUS_sanger/clinical/clinical_stage_annotations.txt",sep="\t",header=T,stringsAsFactors=F)
 all_final_clinic$stage[all_final_clinic$stage == "SMM - progressed"]<- "MGUS-SMM PD"
 all_final_clinic$stage[all_final_clinic$stage == "MGUS - progressed"]<- "MGUS-SMM PD"
 all_final_clinic$stage[all_final_clinic$stage == "SMM stable" ]<- "MGUS-SMM SD"
 all_final_clinic$stage[all_final_clinic$stage == "MGUS" ]<- "MGUS-SMM SD"
 all_final_clinic$stage[all_final_clinic$stage == "MM RR" ]<- "MM"
 all_final_clinic$stage[all_final_clinic$stage == "MM DG" ]<- "MM"
 all_final_clinic<- all_final_clinic[!all_final_clinic$stage %in% c("MM - PCL"),]
 all_final_clinic<- all_final_clinic[!is.na(all_final_clinic$stage),]

#### updated clinical file WXS
 clin<- read.delim("~//Desktop/project/MGUS_sanger/walker_MGUS/results/clinical_MGUS_ProgressionUpdade_03-2017_AM.txt",
                  stringsAsFactors = F)
 colnames(clin)[1]<-"Individual.ID"
 key<- read.delim("~//Desktop/project/MGUS_sanger/walker_MGUS/results/sample_sheet_key.txt", stringsAsFactors = F)
 key2<- key[,c("Experiment.System.ID","Individual.ID")]
 clin_code<- merge(clin, key2)
 colnames(clin_code)[ncol(clin_code)]<-"sample"
 clin_code_2<- clin_code[,c("sample","Stage")]
 clin_code_2$Stage[clin_code_2$Stage == "without_progression" ]<-"MGUS-SMM SD"
 clin_code_2$Stage[clin_code_2$Stage == "PROGRESSION" ]<-"MGUS-SMM PD"
 colnames(clin_code_2)<- colnames(all_final_clinic)[1:2]

### combine WGS and WXS clinical data
 all_final_clinic_w<- rbind.data.frame(all_final_clinic[,1:2], clin_code_2)
 clin<- all_final_clinic_w
```

# 1. Non-progressed dnds analysis

```
 nonPro  = setdiff(clin[which(clin$stage=="MGUS-SMM SD"),"sample"],c("PD47563c"))
 muts_NonPro = muts[which(muts$sample %in% nonPro),]

# All genes:
 d_NonPro = dndscv(muts_NonPro,outmats=T)
```

```
## [1] Loading the environment...
```

```
## [2] Annotating the mutations...
```

```
## [3] Estimating global rates...
```

```
## [4] Running dNdSloc...
```

```
## [5] Running dNdScv...
```

```
##     Regression model for substitutions (theta = 1.7).
```

```
##     Regression model for indels (theta = 0.114)
```

```
 d_NonPro$globaldnds
```

```
##      name   mle  cilow cihigh
## wmis wmis 1.007 0.8789  1.154
## wnon wnon 1.068 0.7747  1.473
## wspl wspl 1.032 0.6847  1.554
## wtru wtru 1.055 0.8097  1.374
## wall wall 1.011 0.8836  1.157
```

## print gene under positive selection

```
    sig_mgus_all<- d_NonPro$sel_cv
    sig_mgus_all[sig_mgus_all$qallsubs_cv<0.1,]
```

```
##      gene_name n_syn n_mis n_non n_spl n_ind wmis_cv wnon_cv wspl_cv wind_cv
## 2151      BTG2     0     0     2     0     1     0.0    7456    7456   302.7
## 8380     IGLL5     3     6     0     0     0   127.6       0       0     0.0
##        pmis_cv    ptrunc_cv    pallsubs_cv  pind_cv    qmis_cv qtrunc_cv
## 2151 9.137e-01 0.0000001002 0.000000675782 0.003252 0.98316245  0.002014
## 8380 2.912e-10 0.9338147388 0.000000002335 1.000000 0.00000585  0.996807
##      qallsubs_cv    pglobal_cv qglobal_cv
## 2151   0.0067886 0.00000004600  0.0004896
## 8380   0.0000469 0.00000004873  0.0004896
```

## Restricted Hypothesis Testing on 81 driver genes in multiple myeloma:

```
    d_NonPro_rht = dndscv(muts_NonPro,gene_list=drivers$gene,outmats=T)
```

```
## [1] Loading the environment...
```

```
## [2] Annotating the mutations...
```

```
## [3] Estimating global rates...
```

```
## [4] Running dNdSloc...
```

```
## [5] Running dNdScv...
```

```
##     Regression model for substitutions (theta = 0.172).
```

```
##     Regression model for indels (theta = 0.565)
```

```
    d_NonPro_rht$globaldnds
```

```
##      name    mle   cilow cihigh
## wmis wmis 0.5780 0.24461  1.366
## wnon wnon 0.8356 0.07560  9.235
## wspl wspl 0.6883 0.07466  6.345
## wtru wtru 0.7490 0.13035  4.303
## wall wall 0.5847 0.24869  1.375
```

```
# print gene under positive selection
    sig_mgus_sel<- d_NonPro_rht$sel_cv
    sig_mgus_sel[sig_mgus_sel$qallsubs_cv<0.1,]
```

```
##  [1] gene_name   n_syn       n_mis       n_non       n_spl       n_ind      
##  [7] wmis_cv     wnon_cv     wspl_cv     wind_cv     pmis_cv     ptrunc_cv  
## [13] pallsubs_cv pind_cv     qmis_cv     qtrunc_cv   qallsubs_cv pglobal_cv 
## [19] qglobal_cv 
## <0 rows> (or 0-length row.names)
```

```
# Function to calculate confidence intervals for dN/dS values per gene under the dNdScv model using profile likelihood.
 mgus_driver_dnds<- geneci(d_NonPro_rht, gene_list = drivers$Gene.Symbol, level = 0.95)
```

```
## Calculating CI95 across all genes...
```

```
 mgus_driver_dnds[mgus_driver_dnds$mis_low>1 | mgus_driver_dnds$tru_low>1,]
```

```
##        gene mis_mle tru_mle mis_low tru_low mis_high tru_high
## 19    DUSP2   5.356   150.9  0.3056   8.613    23.58    664.6
## 27 HIST1H1E  20.187     0.0  3.3461   0.000    52.35  10000.0
```

```
# Site dnds 
 data("knownhotspots_hg19", package="dndscv") # Previously known hotspots
 known_hotspots = known_hotspots[sapply(known_hotspots, function(x) strsplit(x, split=":")[[1]][5]) %in%   drivers$gene] # Known hotspots in targetgenes
 hotspots_siteRHT_81_sd = sitednds(d_NonPro, site_list = known_hotspots, min_recurr = 1, method = "LNP")
```

```
## [1] Site-wise overdispersed model accounting for trinucleotides and relative gene mutability...
```

```
##     Binning the rate vector: maximum deviation of 0.001
```

```
## [2] Calculating site-wise dN/dS ratios and p-values...
```

```
##     Using the conservative bound of the confidence interval of the overdispersion parameter.
```

```
##     Peforming Restricted Hypothesis Testing on the input list of a-priori sites (numtests = length(site_list))
```

```
##     Mutations at known hotspots: 2 observed, 0.00729 expected, obs/exp~274 (CI95:33.2,991).
```

```
##     Modelling substitution rates using a Lognormal-Poisson: sig = 0.01 (upperbound = 0.0963)
```

```
 hotspots_siteRHT_81_sd
```

```
## $recursites
##   chr       pos ref mut gene aachange   impact ref3_cod mut3_cod freq
## 1   1 115258745   C   G NRAS     G13R Missense      TGG      TCG    1
## 2  12  25378562   C   T KRAS    A146T Missense      AGC      AAC    1
##            mu   dnds        pval     qval
## 1 0.000001067 937046 0.000001067 0.000653
## 2 0.000012782  78235 0.000012782 0.003911
## 
## $overdisp
##       MLE CI95_high 
##   0.01000   0.09629 
## 
## $fpr_nonsyn_q05
## NULL
## 
## $LL
## [1] -14453
## 
## $globaldnds_knownsites
##        obs        exp       dnds      cilow     cihigh 
##   2.000000   0.007294 274.198848  33.206753 990.500518
```

# 2. Progressor myeloma precursor disease dnds analysis

```
 progressed  = clin[which(clin$stage=="MGUS-SMM PD"),"sample"]
 muts_progressed = muts[which(muts$sample %in% progressed),]

# All drivergenes:
 d_Prog = dndscv(muts_progressed,outmats=T)
```

```
## [1] Loading the environment...
```

```
## [2] Annotating the mutations...
```

```
## [3] Estimating global rates...
```

```
## [4] Running dNdSloc...
```

```
## [5] Running dNdScv...
```

```
##     Regression model for substitutions (theta = 0.774).
```

```
##     Regression model for indels (theta = 46.7)
```

```
 d_Prog$globaldnds
```

```
##      name    mle  cilow cihigh
## wmis wmis 0.9079 0.7814  1.055
## wnon wnon 0.8322 0.5669  1.221
## wspl wspl 1.0266 0.6518  1.617
## wtru wtru 0.9032 0.6657  1.225
## wall wall 0.9064 0.7816  1.051
```

```
    # gene_cis_smm_pd = geneci(d_Prog,gene_list=drivers$gene)
```

```
# print gene under positive selection
    sig_smm_sel<- d_Prog$sel_cv
    sig_smm_sel[sig_smm_sel$qallsubs_cv<0.1,]
```

```
##       gene_name n_syn n_mis n_non n_spl n_ind wmis_cv wnon_cv wspl_cv wind_cv
## 11632      NRAS     0     4     0     0     0   447.3       0       0       0
##              pmis_cv ptrunc_cv   pallsubs_cv pind_cv    qmis_cv qtrunc_cv
## 11632 0.000000003334    0.9649 0.00000002527       1 0.00006699     0.993
##       qallsubs_cv   pglobal_cv qglobal_cv
## 11632   0.0005077 0.0000004673   0.009389
```

## Restricted Hypothesis Testing on 81 driver genes in multiple myeloma:

```
# RHT:
 d_Prog_rht = dndscv(muts_progressed,gene_list=drivers$gene,outmats=T)
```

```
## [1] Loading the environment...
```

```
## [2] Annotating the mutations...
```

```
## [3] Estimating global rates...
```

```
## [4] Running dNdSloc...
```

```
## [5] Running dNdScv...
```

```
##     Regression model for substitutions (theta = 0.0274).
```

```
 d_Prog_rht$globaldnds
```

```
##      name    mle   cilow cihigh
## wmis wmis 1.9957 0.65773  6.055
## wnon wnon 3.3865 0.53024 21.629
## wspl wspl 0.8084 0.07887  8.285
## wtru wtru 1.7258 0.34690  8.586
## wall wall 2.0082 0.66949  6.024
```

```
    #      name       mle      cilow    cihigh
    # wmis wmis 1.1806792 0.44673959  3.120394
    # wnon wnon 2.3658227 0.40109864 13.954466
    # wspl wspl 0.6154479 0.06272976  6.038219
    # wtru wtru 1.2822099 0.28171292  5.835949
    # wall wall 1.2175518 0.46749364  3.171022
```

```
 sig_smm_sel_rht<- d_Prog_rht$sel_cv
 sig_smm_sel_rht[sig_smm_sel_rht$qallsubs_cv<0.1,]
```

```
##    gene_name n_syn n_mis n_non n_spl wmis_cv wnon_cv wspl_cv   pmis_cv
## 74      TP53     0     0     1     1     0.0   147.8   147.8 0.7181231
## 51      NRAS     0     4     0     0   116.9     0.0     0.0 0.0002907
##    ptrunc_cv pallsubs_cv qmis_cv qtrunc_cv qallsubs_cv
## 74 0.0005229    0.001122 0.87098   0.04183     0.04543
## 51 0.9198968    0.001136 0.02326   1.00000     0.04543
```

```
# Function to calculate confidence intervals for dN/dS values per gene under the dNdScv model using profile likelihood.
 smm_driver_dnds<- geneci(d_Prog_rht, gene_list = drivers$Gene.Symbol, level = 0.95)
```

```
## Calculating CI95 across all genes...
```

```
 smm_driver_dnds[smm_driver_dnds$mis_low>1 | smm_driver_dnds$tru_low>1,]
```

```
##       gene mis_mle tru_mle mis_low tru_low mis_high tru_high
## 4    ARID2    0.00   27.44   0.000   1.566    6.784   120.83
## 16    DIS3   14.08    0.00   1.756   0.000   43.466    67.19
## 39    KRAS   37.57    0.00   2.144   0.000  165.442   307.11
## 51    NRAS  116.87    0.00  16.471   0.000  271.494   379.85
## 54    PIM1   19.10    0.00   1.090   0.000   84.087   223.15
## 74    TP53    0.00  147.84   0.000  17.468   29.482   456.51
## 79 ZFP36L1   18.16    0.00   1.036   0.000   79.962   716.41
```

```
# sitednds:  
 data("knownhotspots_hg19", package="dndscv") # Previously known hotspots
 known_hotspots = known_hotspots[sapply(known_hotspots, function(x) strsplit(x, split=":")[[1]][5]) %in% drivers$gene] # Known hotspots in targetgenes
 hotspots_siteRHT_81_pd = sitednds(d_Prog, site_list = known_hotspots, min_recurr = 1, method = "LNP")
```

```
## [1] Site-wise overdispersed model accounting for trinucleotides and relative gene mutability...
```

```
##     Binning the rate vector: maximum deviation of 0.00108
```

```
## [2] Calculating site-wise dN/dS ratios and p-values...
```

```
##     Using the conservative bound of the confidence interval of the overdispersion parameter.
```

```
##     Peforming Restricted Hypothesis Testing on the input list of a-priori sites (numtests = length(site_list))
```

```
##     Mutations at known hotspots: 8 observed, 0.00765 expected, obs/exp~1.05e+03 (CI95:452,2.06e+03).
```

```
##     Modelling substitution rates using a Lognormal-Poisson: sig = 0.01 (upperbound = 0.099)
```

```
 hotspots_siteRHT_81_pd
```

```
## $recursites
##   chr       pos ref mut gene aachange   impact ref3_cod mut3_cod freq
## 1   1 115256529   T   C NRAS     Q61R Missense      CAA      CGA    2
## 2   7 140453134   T   C BRAF    K601E Missense      GAA      GGA    1
## 3   1 115256530   G   T NRAS     Q61K Missense      ACA      AAA    1
## 4  12  25398284   C   T KRAS     G12D Missense      GGT      GAT    1
## 5  13  73336102   T   G DIS3    L767F Missense      TAG      TCG    1
## 6   1 115258747   C   T NRAS     G12D Missense      GGT      GAT    1
## 7  17   7578275   G   A TP53    Q192* Nonsense      TCA      TTA    1
##            mu   dnds      pval          qval
## 1 0.000007542 265177 2.872e-11 0.00000001758
## 2 0.000006119 163420 6.119e-06 0.00098152146
## 3 0.000006508 153660 6.508e-06 0.00098152146
## 4 0.000007872 127025 7.872e-06 0.00098152146
## 5 0.000008019 124699 8.019e-06 0.00098152146
## 6 0.000010586  94468 1.059e-05 0.00107976605
## 7 0.000014513  68903 1.451e-05 0.00126884135
## 
## $overdisp
##       MLE CI95_high 
##   0.01001   0.09896 
## 
## $fpr_nonsyn_q05
## NULL
## 
## $LL
## [1] -10953
## 
## $globaldnds_knownsites
##         obs         exp        dnds       cilow      cihigh 
##    8.000000    0.007648 1046.086028  451.625698 2061.206500
```

# 3. Multiple myeloma WGS dnds analysis

```
 mm  = clin[which(clin$stage=="MM"),"sample"]
 muts_mm = muts[which(muts$sample %in% mm),]

## create unique catalogue of SNV for patient with more than one sample 
 muts_mm_pd<- muts_mm[grep("PD", muts_mm$sample),]
 muts_mm_no_pd<- muts_mm[-grep("PD", muts_mm$sample),]
 muts_mm_pd[,1] = substr(muts_mm_pd[,1],1,nchar(muts_mm_pd[,1])-1)
 muts_mm_final<- rbind.data.frame(unique(muts_mm_pd), muts_mm_no_pd)

# All genes:
 d_mm = dndscv(muts_mm_final,outmats=T)
```

```
## [1] Loading the environment...
```

```
## [2] Annotating the mutations...
```

```
## [3] Estimating global rates...
```

```
## [4] Running dNdSloc...
```

```
## [5] Running dNdScv...
```

```
##     Regression model for substitutions (theta = 3.41).
```

```
##     Regression model for indels (theta = 68.1)
```

```
 d_mm$globaldnds
```

```
##      name    mle  cilow cihigh
## wmis wmis 1.0234 0.9383  1.116
## wnon wnon 1.0081 0.8278  1.228
## wspl wspl 0.9823 0.7579  1.273
## wtru wtru 0.9989 0.8479  1.177
## wall wall 1.0213 0.9375  1.113
```

```
    # gene_cis_mm_wgs = geneci(d_mm,gene_list=drivers$gene)
```

```
    sig_mm_sel<- d_mm$sel_cv
    sig_mm_sel[sig_mm_sel$qallsubs_cv<0.1,]
```

```
##       gene_name n_syn n_mis n_non n_spl n_ind wmis_cv wnon_cv wspl_cv wind_cv
## 9225       KRAS     0    10     0     0     0  458.68     0.0     0.0       0
## 11632      NRAS     0    10     0     0     0  443.91     0.0     0.0       0
## 2086       BRAF     0     5     0     0     0   54.60     0.0     0.0       0
## 18057      TP53     0     3     0     1     0   58.42   180.3   180.3       0
##           pmis_cv ptrunc_cv pallsubs_cv pind_cv  qmis_cv qtrunc_cv qallsubs_cv
## 9225  0.000000000  0.943660 0.000000000       1 0.000000    0.9811     0.00000
## 11632 0.000000000  0.944205 0.000000000       1 0.000000    0.9811     0.00000
## 2086  0.000001013  0.871069 0.000006199       1 0.006781    0.9811     0.04151
## 18057 0.000067369  0.004583 0.000012682       1 0.169189    0.9811     0.06370
##       pglobal_cv qglobal_cv
## 9225  0.00000000     0.0000
## 11632 0.00000000     0.0000
## 2086  0.00008053     0.4045
## 18057 0.00015567     0.6255
```

## Restricted Hypothesis Testing on 81 driver genes in multiple myeloma:

```
# 81 drivers:   
    d_mm_rht = dndscv(muts_mm_final,outmats=T,gene_list=drivers$gene)
```

```
## [1] Loading the environment...
```

```
## [2] Annotating the mutations...
```

```
## [3] Estimating global rates...
```

```
## [4] Running dNdSloc...
```

```
## [5] Running dNdScv...
```

```
##     Regression model for substitutions (theta = 0.154).
```

```
##     Regression model for indels (theta = 0.259)
```

```
    d_mm_rht$globaldnds
```

```
##      name   mle  cilow cihigh
## wmis wmis 3.121 1.4028  6.943
## wnon wnon 3.440 1.0565 11.201
## wspl wspl 1.430 0.2718  7.528
## wtru wtru 2.556 0.8735  7.480
## wall wall 3.084 1.3929  6.828
```

```
    sig_mm_sel_rht<- d_mm_rht$sel_cv
    sig_mm_sel_rht[sig_mm_sel_rht$qallsubs_cv<0.1,]
```

```
##    gene_name n_syn n_mis n_non n_spl n_ind wmis_cv wnon_cv wspl_cv wind_cv
## 39      KRAS     0    10     0     0     0   159.1       0       0       0
## 51      NRAS     0    10     0     0     0   147.2       0       0       0
##          pmis_cv ptrunc_cv   pallsubs_cv pind_cv     qmis_cv qtrunc_cv
## 39 0.00000001289    0.8981 0.00000006917       1 0.000001032     0.979
## 51 0.00000002631    0.8974 0.00000013801       1 0.000001052     0.979
##    qallsubs_cv  pglobal_cv qglobal_cv
## 39 0.000005521 0.000001210 0.00009272
## 51 0.000005521 0.000002318 0.00009272
```

```
# Function to calculate confidence intervals for dN/dS values per gene under the dNdScv model using profile likelihood.
 mm_driver_dnds<- geneci(d_mm_rht, gene_list = drivers$Gene.Symbol, level = 0.95)
```

```
## Calculating CI95 across all genes...
```

```
 mm_driver_dnds[mm_driver_dnds$mis_low>1 | mm_driver_dnds$tru_low>1,]
```

```
##      gene mis_mle tru_mle mis_low tru_low mis_high tru_high
## 2   ACTG1  17.606    0.00  2.3549   0.000   54.363   178.99
## 9    BRAF  20.539    0.00  4.0524   0.000   44.144    51.76
## 13 CDKN2C  18.224    0.00  1.0398   0.000   80.239   558.87
## 15   CYLD   3.306   46.79  0.1886   6.554   14.557   144.49
## 16   DIS3  12.978    0.00  2.4797   0.000   30.149    42.36
## 22 FAM46C  17.391    0.00  2.3280   0.000   53.701   276.38
## 33   IRF4  14.836    0.00  1.9824   0.000   45.812   107.03
## 39   KRAS 159.127    0.00 31.6124   0.000  279.098   234.34
## 45 MAN2C1   0.000   19.26  0.0000   1.099    6.363    84.81
## 51   NRAS 147.165    0.00 29.0628   0.000  258.119   231.04
## 55   POT1   9.129    0.00  1.2521   0.000   28.188    64.84
## 56  PRDM1   7.929    0.00  1.1008   0.000   24.482    96.65
## 59  RASA2   0.000   21.03  0.0000   1.200    6.987    92.61
## 64 RPRD1B  33.064    0.00  1.5861   0.000   85.738   107.83
## 68  SF3B1   7.117    0.00  1.2780   0.000   18.456    32.31
## 74   TP53  26.729   50.13  4.3163   2.860   69.311   220.71
## 78   XBP1   0.000   74.26  0.0000   4.237   18.181   326.99
```

```
    data("knownhotspots_hg19", package="dndscv") # Previously known hotspots
    known_hotspots = known_hotspots[sapply(known_hotspots, function(x) strsplit(x, split=":")[[1]][5]) %in% drivers$gene] # Known hotspots in targetgenes
    hotspots_siteRHT_81_mm = sitednds(d_mm, site_list = known_hotspots, min_recurr = 1, method = "LNP")
```

```
## [1] Site-wise overdispersed model accounting for trinucleotides and relative gene mutability...
```

```
##     Binning the rate vector: maximum deviation of 0.00024
```

```
## [2] Calculating site-wise dN/dS ratios and p-values...
```

```
##     Using the conservative bound of the confidence interval of the overdispersion parameter.
```

```
##     Peforming Restricted Hypothesis Testing on the input list of a-priori sites (numtests = length(site_list))
```

```
##     Mutations at known hotspots: 27 observed, 0.025 expected, obs/exp~1.08e+03 (CI95:710,1.57e+03).
```

```
##     Modelling substitution rates using a Lognormal-Poisson: sig = 0.01 (upperbound = 0.0953)
```

```
    hotspots_siteRHT_81_mm
```

```
## $recursites
##    chr       pos ref mut   gene aachange   impact ref3_cod mut3_cod freq
## 1    1 115256529   T   C   NRAS     Q61R Missense      CAA      CGA    6
## 2   12  25380275   T   G   KRAS     Q61H Missense      AAG      ACG    3
## 3   12  25378562   C   T   KRAS    A146T Missense      AGC      AAC    2
## 4   12  25398281   C   T   KRAS     G13D Missense      GGC      GAC    2
## 5    7 140453136   A   T   BRAF    V600E Missense      GTG      GAG    1
## 6    7 140453134   T   C   BRAF    K601E Missense      GAA      GGA    1
## 7    1 115256528   T   A   NRAS     Q61H Missense      AAG      ATG    1
## 8   17   7577114   C   G   TP53    C275S Missense      TGT      TCT    1
## 9   12 112926888   G   T PTPN11    G503V Missense      GGG      GTG    1
## 10  17   7577551   C   T   TP53    G244S Missense      GGG      GAG    1
## 11  13  73336102   T   G   DIS3    L767F Missense      TAG      TCG    1
## 12   1 115256530   G   T   NRAS     Q61K Missense      ACA      AAA    1
## 13   7 140453154   T   C   BRAF    D594G Missense      GAT      GGT    1
## 14  12  25378647   T   A   KRAS    K117N Missense      AAT      ATT    1
## 15  17   7577097   C   G   TP53    D281H Missense      AGA      ACA    1
## 16   1 115258744   C   T   NRAS     G13D Missense      GGT      GAT    1
## 17   1 115258747   C   T   NRAS     G12D Missense      GGT      GAT    1
## 18  12  25398284   C   T   KRAS     G12D Missense      GGT      GAT    1
##             mu   dnds      pval      qval
## 1  0.000023109 259636 2.424e-31 1.484e-28
## 2  0.000038173  78589 9.527e-15 2.915e-12
## 3  0.000048875  40921 1.205e-09 2.459e-07
## 4  0.000062673  31912 1.982e-09 3.032e-07
## 5  0.000008812 113479 8.812e-06 1.079e-03
## 6  0.000012406  80608 1.241e-05 1.265e-03
## 7  0.000015990  62538 1.599e-05 1.301e-03
## 8  0.000017004  58809 1.700e-05 1.301e-03
## 9  0.000019948  50129 1.995e-05 1.356e-03
## 10 0.000024973  40043 2.497e-05 1.423e-03
## 11 0.000025573  39104 2.557e-05 1.423e-03
## 12 0.000030504  32782 3.050e-05 1.447e-03
## 13 0.000032400  30865 3.240e-05 1.447e-03
## 14 0.000035363  28278 3.536e-05 1.447e-03
## 15 0.000035664  28039 3.566e-05 1.447e-03
## 16 0.000042547  23504 4.255e-05 1.447e-03
## 17 0.000042547  23504 4.255e-05 1.447e-03
## 18 0.000042547  23504 4.255e-05 1.447e-03
## 
## $overdisp
##       MLE CI95_high 
##   0.01004   0.09535 
## 
## $fpr_nonsyn_q05
## NULL
## 
## $LL
## [1] -32041
## 
## $globaldnds_knownsites
##        obs        exp       dnds      cilow     cihigh 
##   27.00000    0.02505 1077.87395  710.32573 1568.25001
```

# 4. Multiple myeloma in CoMMpass trial dnds analysis

```
    commpass<- read.delim("~/Desktop/project/MGUS_sanger/walker_MGUS/MMRF_CoMMpass_IA15a_All_Canonical_Variants.txt",
                          stringsAsFactors = F)
    commpass2<- commpass[,c(1,2,3,5,6)]
    commpass2<- commpass2[grep("1_BM", commpass2$Sample),]
    commpass2<- unique(commpass2)
    colnames(commpass2)<-c( "sample", "chr","pos","ref","alt"  )
    length(unique(commpass2$sample))
```

```
## [1] 947
```

```
    # All genes:
    commpass_mm = dndscv(commpass2,outmats=T)
```

```
## [1] Loading the environment...
```

```
## [2] Annotating the mutations...
```

```
##     Note: 238 mutations removed for exceeding the limit of mutations per gene per sample (see the max_muts_per_gene_per_sample argument in dndscv)
```

```
##     14% ...
```

```
##     28% ...
```

```
##     42% ...
```

```
##     56% ...
```

```
##     70% ...
```

```
##     84% ...
```

```
##     98% ...
```

```
## [3] Estimating global rates...
```

```
## [4] Running dNdSloc...
```

```
## [5] Running dNdScv...
```

```
##     Regression model for substitutions (theta = 4.77).
```

```
##     Regression model for indels (theta = 0.847)
```

```
    commpass_mm$globaldnds
```

```
##      name    mle  cilow cihigh
## wmis wmis 1.0391 1.0209  1.058
## wnon wnon 1.0313 0.9909  1.073
## wspl wspl 0.9441 0.8900  1.001
## wtru wtru 1.0038 0.9700  1.039
## wall wall 1.0364 1.0185  1.055
```

```
    # gene_cis_commpass = geneci(commpass_mm,gene_list=drivers$gene)
```

```
    sig_mm_comm_sel<- commpass_mm$sel_cv
    sig_mm_comm_sel[sig_mm_comm_sel$qallsubs_cv<0.1,]
```

```
##       gene_name n_syn n_mis n_non n_spl n_ind  wmis_cv wnon_cv wspl_cv wind_cv
## 18125     TRAF3     2    39    25     4    23  20.5575 156.286 156.286 262.850
## 10192       MAX     0    28     5     1     0  60.0856  97.224  97.224   0.000
## 18057      TP53     1    42     2     3     4  31.8893  35.712  35.712  89.018
## 5076       DIS3     1   103     0     5     0  43.8336  16.393  16.393   0.000
## 6238     FAM46C     5    51     3     0    44  24.2700  27.304  27.304 818.224
## 8380      IGLL5   105   169     1     8     9   7.8524   4.598   4.598 199.425
## 2086       BRAF     1    68     0     0     0  33.0292   0.000   0.000   0.000
## 9225       KRAS     1   264     0     0     1 525.0631   0.000   0.000  31.836
## 11632      NRAS     1   217     0     0     0 423.9082   0.000   0.000   0.000
## 5385      DUSP2    22    37     5     9     3   4.9811  41.878  41.878  33.204
## 9897        LTB    13    25     1    11     0   5.7981  43.590  43.590   0.000
## 1947      BCL7A     6    14     0    11     2   9.8338  60.538  60.538  93.036
## 4667       CYLD     0    11    12     2     6   5.9132  64.446  64.446  27.750
## 7872   HIST1H1E     4    42     0     0     1  14.3542   0.000   0.000   6.243
## 16476     SP140     2    11     9     3     7   3.4484  26.120  26.120  48.054
## 17302     TCL1A     5    20     0     1     1  20.0605   8.629   8.629  75.476
## 17205   TBC1D29     2    19     0     0     0  33.7218   0.000   0.000   0.000
## 18124     TRAF2     0     7     8     2     1   6.3052 110.881 110.881  14.734
## 11342    NFKBIA     0     5     7     0     6   4.8613  60.433  60.433  38.603
## 5557       EGR1    16    38     0     0     4   4.8092   0.000   0.000  40.117
## 6523      FGFR3     4    26     0     0     4   6.2602   0.000   0.000  69.803
## 13629     PRKD2     1    28     0     0     1  13.1455   0.000   0.000   8.495
## 12403    PABPC1     5    28     0     0     2   8.2208   0.000   0.000  14.894
## 15276    SAMHD1     0    19     2     3     1  13.9290  26.391  26.391   8.915
## 14249       RB1     0     3     6     2     4   1.5959  27.125  27.125  23.207
## 13920    PTPN11     0    22     0     0     0  17.3676   0.000   0.000   0.000
## 9169      KLHL6     4    26     1     3     0   8.3751  15.810  15.810   0.000
## 6773      FUBP1     1     7     2     5     3   4.1627  26.170  26.170  22.224
## 8620       IRF4     2    20     0     0     0  12.8103   0.000   0.000   0.000
## 7869   HIST1H1B     4    14     0     0     3   7.4765   0.000   0.000  41.134
## 14211     RASA2     0     8     3     2     4   4.9427  21.582  21.582  25.082
## 3271      CCND1    17    27     0     0     0   5.5691   0.000   0.000   0.000
## 17437      TGDS     0     9     1     1     2  12.5356  19.818  19.818  34.098
## 13556     PRDM1     3    15     3     0     3   4.5463  13.545  13.545  21.849
## 516       ACTG1     8    28     2     0     1   5.1236   5.721   5.721   5.110
## 12991      PIM1     4    19     1     0     0   8.7151   5.333   5.333   0.000
## 11341     NFKB2     5    23     4     0     1   5.5756   8.634   8.634   5.607
## 14991     RPL10     2    14     0     0     0  10.3150   0.000   0.000   0.000
## 2150       BTG1     7    14     3     1     0   5.1655  16.208  16.208   0.000
## 8614       IRF1     0     6     4     0     0   8.1197  46.542  46.542   0.000
## 11007     MYO1E     2     7     0     7     1   2.0869  15.733  15.733   8.005
## 9205      KMT2B     4    10     7     1     1   1.2548  12.533  12.533   2.988
## 1467      ARID2     3     4     6     1     2   0.7054   9.996   9.996   6.382
## 14431     RFTN1     6    10     2     4     0   3.1281  18.415  18.415   0.000
## 13336      POT1     0    13     0     1     1   9.4952   4.926   4.926   8.747
## 5563       EHD1     2    15     0     0     1   7.2785   0.000   0.000   6.035
## 14256     RBBP7     0     2     1     3     1   2.0160  31.359  31.359  17.636
## 4293     CREBBP     3     8     5     2     2   1.2902  10.685  10.685   5.527
## 5752      EP300     3     5     5     2     2   0.7968   8.928   8.928   5.389
## 5360       DTX1     5    18     0     0     1   4.8127   0.000   0.000  11.604
## 15086     RPS3A     0    10     0     0     0  12.5221   0.000   0.000   0.000
## 12465     PANK3     1    10     0     1     0  10.1313   8.873   8.873   0.000
## 18245    TRIM73     7     7     0     0     0  12.1147   0.000   0.000   0.000
## 4888      DDX3X     1    15     0     3     0   6.2586  12.335  12.335   0.000
## 7871   HIST1H1D     4    15     0     0     0   6.3387   0.000   0.000   0.000
## 12037     OR2L8     0     9     0     0     0  12.0933   0.000   0.000   0.000
## 8313     IFNA16     0     0     3     0     0   0.0000  51.866  51.866   0.000
## 16053   SLC35G5     0    12     0     0     0   9.4845   0.000   0.000   0.000
## 2786    C8orf86     0     3     3     0     0   4.7103  53.461  53.461   0.000
## 2036      BMP2K     0    15     3     0     0   6.7869  11.236  11.236   0.000
## 9604     LILRA6     1    11     1     0     0   8.2914   6.996   6.996   0.000
## 9614      LIMD2     6     7     1     0     0   8.4407  13.329  13.329   0.000
## 15321     SCAF8     2     4     4     2     0   1.0380  12.650  12.650   0.000
##         pmis_cv ptrunc_cv pallsubs_cv   pind_cv   qmis_cv qtrunc_cv qallsubs_cv
## 18125 0.000e+00 0.000e+00   0.000e+00 1.226e-24 0.000e+00 0.000e+00   0.000e+00
## 10192 0.000e+00 1.426e-09   0.000e+00 1.000e+00 0.000e+00 3.582e-06   0.000e+00
## 18057 0.000e+00 2.320e-06   0.000e+00 4.624e-06 0.000e+00 3.885e-03   0.000e+00
## 5076  0.000e+00 9.829e-05   0.000e+00 1.000e+00 0.000e+00 7.595e-02   0.000e+00
## 6238  0.000e+00 2.786e-04   0.000e+00 6.899e-55 0.000e+00 1.866e-01   0.000e+00
## 8380  0.000e+00 3.219e-04   0.000e+00 1.386e-12 0.000e+00 2.021e-01   0.000e+00
## 2086  0.000e+00 4.477e-01   0.000e+00 1.000e+00 0.000e+00 8.767e-01   0.000e+00
## 9225  0.000e+00 7.337e-01   0.000e+00 3.037e-02 0.000e+00 8.767e-01   0.000e+00
## 11632 0.000e+00 7.517e-01   0.000e+00 1.000e+00 0.000e+00 8.767e-01   0.000e+00
## 5385  4.577e-10 0.000e+00   0.000e+00 6.721e-04 6.131e-07 0.000e+00   0.000e+00
## 9897  2.930e-08 7.438e-15   0.000e+00 1.000e+00 2.676e-05 3.736e-11   0.000e+00
## 1947  1.094e-07 1.099e-14   0.000e+00 4.803e-04 9.156e-05 4.416e-11   0.000e+00
## 4667  1.127e-03 1.554e-15   1.232e-14 4.906e-05 3.019e-01 1.041e-11   1.651e-11
## 7872  0.000e+00 7.379e-01   3.331e-16 1.365e-01 0.000e+00 8.767e-01   5.147e-13
## 16476 1.271e-02 1.486e-10   1.083e-09 9.854e-07 8.604e-01 4.264e-07   1.088e-06
## 17302 2.920e-14 1.170e-01   1.882e-13 1.306e-02 4.889e-11 8.767e-01   2.224e-10
## 17205 4.441e-16 7.137e-01   3.331e-15 1.000e+00 8.111e-13 8.767e-01   4.780e-12
## 18124 3.135e-03 1.465e-14   7.150e-14 6.320e-02 6.115e-01 4.907e-11   8.978e-11
## 11342 2.213e-02 2.603e-09   1.212e-08 9.565e-06 8.604e-01 5.811e-06   9.740e-06
## 5557  4.690e-09 4.012e-01   1.692e-08 8.896e-05 5.235e-06 8.767e-01   1.308e-05
## 6523  5.554e-07 4.070e-01   1.355e-06 1.159e-05 4.299e-04 8.767e-01   8.783e-04
## 13629 8.489e-11 5.496e-01   3.003e-10 1.044e-01 1.312e-07 8.767e-01   3.352e-07
## 12403 1.383e-09 3.892e-01   3.493e-09 1.486e-02 1.635e-06 8.767e-01   3.051e-06
## 15276 1.356e-08 1.467e-05   1.207e-09 9.998e-02 1.297e-05 1.733e-02   1.155e-06
## 14249 5.510e-01 1.361e-07   5.784e-07 5.963e-04 8.999e-01 2.734e-04   4.007e-04
## 13920 1.205e-10 5.905e-01   4.726e-10 1.000e+00 1.729e-07 8.767e-01   4.997e-07
## 9169  5.578e-09 3.012e-04   1.795e-09 1.000e+00 5.898e-06 1.952e-01   1.639e-06
## 6773  1.591e-02 3.935e-07   1.302e-06 1.960e-03 8.604e-01 7.188e-04   8.720e-04
## 8620  1.057e-09 5.753e-01   4.767e-09 1.000e+00 1.328e-06 8.767e-01   3.990e-06
## 7869  4.913e-06 7.589e-01   2.640e-05 3.732e-04 3.403e-03 8.767e-01   1.326e-02
## 14211 7.398e-03 3.828e-05   5.503e-05 4.591e-04 8.604e-01 4.273e-02   2.571e-02
## 3271  8.317e-09 4.071e-01   3.251e-08 1.000e+00 8.355e-06 8.767e-01   2.419e-05
## 17437 1.448e-05 6.746e-03   1.051e-05 3.303e-03 8.559e-03 8.767e-01   5.559e-03
## 13556 5.744e-04 2.560e-03   1.546e-04 2.049e-03 1.903e-01 8.767e-01   5.548e-02
## 516   5.777e-07 6.285e-02   2.050e-06 1.614e-01 4.299e-04 8.767e-01   1.287e-03
## 12991 6.260e-08 2.000e-01   3.645e-07 1.000e+00 5.468e-05 8.767e-01   2.615e-04
## 11341 3.102e-06 2.755e-03   2.467e-06 1.494e-01 2.226e-03 8.767e-01   1.502e-03
## 14991 5.740e-07 6.043e-01   2.568e-06 1.000e+00 4.299e-04 8.767e-01   1.518e-03
## 2150  6.912e-05 2.234e-04   2.651e-06 1.000e+00 3.308e-02 1.548e-01   1.522e-03
## 8614  1.548e-03 8.448e-06   2.727e-06 1.000e+00 3.747e-01 1.061e-02   1.522e-03
## 11007 1.905e-01 7.022e-06   4.132e-05 1.100e-01 8.604e-01 9.405e-03   1.977e-02
## 9205  6.339e-01 5.607e-06   2.009e-05 2.458e-01 9.230e-01 8.046e-03   1.035e-02
## 1467  5.753e-01 8.758e-05   8.643e-05 5.883e-02 9.063e-01 7.331e-02   3.473e-02
## 14431 1.328e-02 4.862e-06   7.334e-06 1.000e+00 8.604e-01 7.513e-03   3.982e-03
## 13336 1.415e-05 2.293e-01   7.521e-05 1.017e-01 8.559e-03 8.767e-01   3.188e-02
## 5563  1.290e-05 6.536e-01   5.654e-05 1.404e-01 8.284e-03 8.767e-01   2.582e-02
## 14256 4.429e-01 4.063e-05   2.202e-04 5.341e-02 8.755e-01 4.297e-02   7.267e-02
## 4293  6.249e-01 5.842e-05   2.206e-04 7.257e-02 9.215e-01 5.609e-02   7.267e-02
## 5752  6.987e-01 1.722e-04   2.129e-04 7.521e-02 9.436e-01 1.235e-01   7.249e-02
## 5360  6.916e-05 5.164e-01   2.320e-04 7.879e-02 3.308e-02 8.767e-01   7.516e-02
## 15086 7.115e-06 6.965e-01   3.327e-05 1.000e+00 4.765e-03 8.767e-01   1.631e-02
## 12465 1.718e-05 1.184e-01   5.815e-05 1.000e+00 9.590e-03 8.767e-01   2.596e-02
## 18245 1.319e-05 7.378e-01   6.917e-05 1.000e+00 8.284e-03 8.767e-01   3.021e-02
## 4888  1.137e-04 4.043e-03   7.616e-05 1.000e+00 5.077e-02 8.767e-01   3.188e-02
## 7871  1.661e-05 7.178e-01   8.123e-05 1.000e+00 9.537e-03 8.767e-01   3.331e-02
## 12037 1.881e-05 7.522e-01   9.193e-05 1.000e+00 1.021e-02 8.767e-01   3.622e-02
## 8313  2.302e-01 6.433e-05   9.511e-05 1.000e+00 8.604e-01 5.875e-02   3.675e-02
## 16053 2.232e-05 7.255e-01   1.027e-04 1.000e+00 1.180e-02 8.767e-01   3.893e-02
## 2786  6.193e-02 5.863e-05   1.323e-04 1.000e+00 8.604e-01 5.609e-02   4.921e-02
## 2036  1.396e-04 6.121e-03   1.504e-04 1.000e+00 5.947e-02 8.767e-01   5.494e-02
## 9604  4.719e-05 1.537e-01   1.784e-04 1.000e+00 2.431e-02 8.767e-01   6.288e-02
## 9614  1.421e-04 7.162e-02   2.109e-04 1.000e+00 5.947e-02 8.767e-01   7.249e-02
## 15321 9.544e-01 8.124e-05   2.376e-04 1.000e+00 9.941e-01 7.097e-02   7.577e-02
##       pglobal_cv qglobal_cv
## 18125  0.000e+00  0.000e+00
## 10192  0.000e+00  0.000e+00
## 18057  0.000e+00  0.000e+00
## 5076   0.000e+00  0.000e+00
## 6238   0.000e+00  0.000e+00
## 8380   0.000e+00  0.000e+00
## 2086   0.000e+00  0.000e+00
## 9225   0.000e+00  0.000e+00
## 11632  0.000e+00  0.000e+00
## 5385   0.000e+00  0.000e+00
## 9897   0.000e+00  0.000e+00
## 1947   0.000e+00  0.000e+00
## 4667   0.000e+00  0.000e+00
## 7872   1.776e-15  2.549e-12
## 16476  3.786e-14  5.071e-11
## 17302  8.515e-14  1.069e-10
## 17205  1.144e-13  1.351e-10
## 18124  1.538e-13  1.716e-10
## 11342  3.569e-12  3.774e-09
## 5557   4.249e-11  4.268e-08
## 6523   4.065e-10  3.889e-07
## 13629  7.894e-10  7.209e-07
## 12403  1.281e-09  1.119e-06
## 15276  2.877e-09  2.409e-06
## 14249  7.860e-09  6.316e-06
## 13920  1.062e-08  8.206e-06
## 9169   3.794e-08  2.823e-05
## 6773   5.305e-08  3.806e-05
## 8620   9.611e-08  6.658e-05
## 7869   1.915e-07  1.283e-04
## 14211  4.673e-07  3.029e-04
## 3271   5.931e-07  3.611e-04
## 17437  6.311e-07  3.729e-04
## 13556  5.058e-06  2.903e-03
## 516    5.268e-06  2.940e-03
## 12991  5.768e-06  3.082e-03
## 11341  5.829e-06  3.082e-03
## 14991  3.563e-05  1.835e-02
## 2150   3.669e-05  1.843e-02
## 8614   3.766e-05  1.846e-02
## 11007  6.046e-05  2.825e-02
## 9205   6.526e-05  2.980e-02
## 1467   6.706e-05  2.994e-02
## 14431  9.404e-05  4.020e-02
## 13336  9.776e-05  4.092e-02
## 5563   1.012e-04  4.149e-02
## 14256  1.453e-04  5.837e-02
## 4293   1.928e-04  7.309e-02
## 5752   1.928e-04  7.309e-02
## 5360   2.177e-04  8.099e-02
## 15086  3.764e-04  1.304e-01
## 12465  6.252e-04  2.026e-01
## 18245  7.318e-04  2.297e-01
## 4888   7.983e-04  2.410e-01
## 7871   8.463e-04  2.429e-01
## 12037  9.464e-04  2.678e-01
## 8313   9.759e-04  2.723e-01
## 16053  1.046e-03  2.878e-01
## 2786   1.314e-03  3.383e-01
## 2036   1.474e-03  3.656e-01
## 9604   1.718e-03  4.200e-01
## 9614   1.996e-03  4.662e-01
## 15321  2.220e-03  5.012e-01
```

## Restricted Hypothesis Testing on 81 driver genes in multiple myeloma:

```
    commpass_mm_rht = dndscv(commpass2,outmats=T,gene_list=drivers$gene)
```

```
## [1] Loading the environment...
```

```
## [2] Annotating the mutations...
```

```
##     Note: 38 mutations removed for exceeding the limit of mutations per gene per sample (see the max_muts_per_gene_per_sample argument in dndscv)
```

```
## [3] Estimating global rates...
```

```
## [4] Running dNdSloc...
```

```
## [5] Running dNdScv...
```

```
##     Regression model for substitutions (theta = 0.451).
```

```
##     No gene was excluded from the background indel model.
```

```
##     Regression model for indels (theta = 0.385)
```

```
    commpass_mm_rht$globaldnds
```

```
##      name   mle cilow cihigh
## wmis wmis 2.292 2.009  2.616
## wnon wnon 3.397 2.744  4.204
## wspl wspl 2.341 1.823  3.006
## wtru wtru 2.908 2.425  3.488
## wall wall 2.362 2.072  2.692
```

```
    sig_mm_comm_rht_sel<- commpass_mm_rht$sel_cv
    sig_mm_comm_rht_sel[sig_mm_comm_rht_sel$qallsubs_cv<0.1,]
```

```
##    gene_name n_syn n_mis n_non n_spl n_ind   wmis_cv  wnon_cv  wspl_cv wind_cv
## 39      KRAS     1   264     0     0     1 136.84129  0.00000  0.00000  2.0858
## 51      NRAS     1   217     0     0     0 103.55688  0.00000  0.00000  0.0000
## 76     TRAF3     2    39    25     4    23   6.47177 39.13000 39.13000 16.0191
## 16      DIS3     1   103     0     5     0  10.56437  3.70317  3.70317  0.0000
## 1      ABCF1    28     2     1     0     0   0.03044  0.09085  0.09085  0.0000
## 22    FAM46C     5    51     3     0    44   5.87346  6.71530  6.71530 44.4825
## 9       BRAF     1    68     0     0     0   8.88744  0.00000  0.00000  0.0000
## 15      CYLD     0    11    12     2     6   1.16799 11.45430 11.45430  2.4846
## 75     TRAF2     0     7     8     2     1   1.19649 15.64551 15.64551  0.7153
## 74      TP53     1    42     2     3     4  10.61326  8.84723  8.84723  4.0233
## 50    NFKBIA     0     5     7     0     6   1.59362 17.21816 17.21816  7.4773
## 46       MAX     0    28     5     1     0  15.54951 23.65239 23.65239  0.0000
## 21     EP300     3     5     5     2     2   0.20540  2.59793  2.59793  0.3282
## 19     DUSP2    22    37     5     9     3   1.00404  5.19417  5.19417  3.7743
## 69     SP140     2    11     9     3     7   1.21629  7.14013  7.14013  3.1960
## 4      ARID2     3     4     6     1     2   0.22086  3.55214  3.55214  0.4317
## 7      BCL7A     6    14     0    11     2   1.30371  7.83317  7.83317  3.4164
## 27  HIST1H1E     4    42     0     0     1   6.47209  0.00000  0.00000  1.8014
## 41       LTB    13    25     1    11     0   1.39560  7.85079  7.85079  0.0000
## 37     KMT2B     4    10     7     1     1   0.35954  2.76348  2.76348  0.1459
## 14    CREBBP     3     8     5     2     2   0.32492  2.58662  2.58662  0.3244
## 60       RB1     0     3     6     2     4   0.33590  4.94654  4.94654  1.7063
## 72      TET2     2     5     4     0     4   0.24768  2.25353  2.25353  0.7832
## 48       NF1     4    10     1     2     4   0.35002  0.78842  0.78842  0.5582
## 57     PRKD2     1    28     0     0     1   3.03816  0.00000  0.00000  0.4458
## 3     ARID1A     2     7     1     1     4   0.29964  0.94091  0.94091  0.6934
## 35     KDM6A     2     2     2     0     1   0.14270  0.99730  0.99730  0.2813
## 47     NCOR1     2     8     3     2     1   0.32610  1.55454  1.55454  0.1624
## 24     FUBP1     1     7     2     5     3   0.99287  6.18529  6.18529  1.7851
## 58    PTPN11     0    22     0     0     0   3.49450  0.00000  0.00000  0.0000
## 70   TBC1D29     2    19     0     0     0   8.79670  0.00000  0.00000  0.0000
## 80    ZNF292     2    13     6     0     4   0.48626  2.69046  2.69046  0.5819
## 23     FGFR3     4    26     0     0     4   2.92335  0.00000  0.00000  1.9595
## 53    PIK3CA     3     2     0     0     0   0.18699  0.00000  0.00000  0.0000
## 38     KMT2C     5    27     1     2     0   0.54632  0.51221  0.51221  0.0000
## 79   ZFP36L1     3     3     0     3     1   0.66582 12.90534 12.90534  1.1690
## 34     KDM5C     0     5     1     0     1   0.31028  0.51418  0.51418  0.2539
## 66    SAMHD1     0    19     2     3     1   2.90392  4.72132  4.72132  0.6321
## 77      UBR5     2    13     4     1     1   0.45510  1.25555  1.25555  0.1415
## 12    CDKN1B     0     6     2     0     2   2.68379 10.59382 10.59382  3.8476
## 52    PABPC1     5    28     0     0     2   2.32678  0.00000  0.00000  1.2443
## 33      IRF4     2    20     0     0     0   4.20992  0.00000  0.00000  0.0000
## 71     TCL1A     5    20     0     1     1   3.46671  1.23984  1.23984  3.4461
## 67     SETD2     6    18     6     1     5   0.69914  2.79830  2.79830  0.7725
## 59     RASA2     0     8     3     2     4   0.95280  3.65474  3.65474  1.8627
## 6       ATRX     1    14     1     0     0   0.53718  0.31023  0.31023  0.0000
## 61     RFTN1     6    10     2     4     0   0.84126  4.45186  4.45186  0.0000
## 32      IRF1     0     6     4     0     0   1.83955  7.19665  7.19665  0.0000
## 62     RPL10     2    14     0     0     0   4.84303  0.00000  0.00000  0.0000
## 36     KLHL6     4    26     1     3     0   3.37945  5.51223  5.51223  0.0000
## 65     RPS3A     0    10     0     0     0   3.36312  0.00000  0.00000  0.0000
## 11     CCND1    17    27     0     0     0   0.90369  0.00000  0.00000  0.0000
##      pmis_cv     ptrunc_cv pallsubs_cv       pind_cv   qmis_cv    qtrunc_cv
## 39 0.000e+00 0.46348143169   0.000e+00 0.26760090059 0.000e+00 0.6179752423
## 51 0.000e+00 0.47692927134   0.000e+00 1.00000000000 0.000e+00 0.6254810116
## 76 1.694e-05 0.00000000000   0.000e+00 0.00063596134 1.232e-04 0.0000000000
## 16 1.977e-13 0.01681967161   3.442e-13 1.00000000000 4.314e-12 0.0591286464
## 1  2.157e-13 0.00044044005   6.477e-13 1.00000000000 4.314e-12 0.0035235204
## 22 4.959e-06 0.02293067332   2.268e-05 0.00000004648 4.408e-05 0.0733781546
## 9  8.759e-10 0.13401828583   7.353e-12 1.00000000000 1.401e-08 0.2680365717
## 15 6.156e-01 0.00000001496   1.007e-08 0.14248075838 7.243e-01 0.0000005986
## 75 6.449e-01 0.00000006386   1.106e-07 0.44578452967 7.370e-01 0.0000017029
## 74 2.067e-07 0.00142254464   1.285e-06 0.08499470858 2.757e-06 0.0087541209
## 50 3.335e-01 0.00000423914   1.464e-05 0.02328409911 4.601e-01 0.0000678262
## 46 3.717e-07 0.00001266032   4.896e-07 1.00000000000 4.248e-06 0.0001571650
## 21 1.744e-06 0.02921498330   1.009e-06 0.54066402950 1.744e-05 0.0853106712
## 19 9.882e-01 0.00001375194   2.150e-05 0.10309513574 9.882e-01 0.0001571650
## 69 5.294e-01 0.00002811631   2.816e-05 0.10130510512 6.618e-01 0.0002811631
## 4  5.882e-05 0.00587359322   6.698e-06 0.49564100089 3.137e-04 0.0293679661
## 7  5.926e-01 0.00004910583   6.439e-05 0.13677649818 7.229e-01 0.0004364963
## 27 1.534e-05 0.52170221816   3.349e-05 0.29089735156 1.227e-04 0.6466081207
## 41 3.285e-01 0.00000256859   1.152e-05 1.00000000000 4.601e-01 0.0000513718
## 37 9.836e-05 0.01387765342   2.465e-05 0.67689584550 4.212e-04 0.0591286464
## 14 9.385e-05 0.02985873491   4.600e-05 0.54249572590 4.212e-04 0.0853106712
## 60 2.112e-02 0.00063156400   1.156e-04 0.21823587219 4.828e-02 0.0045931927
## 72 5.079e-05 0.15220835608   9.765e-05 0.36782753678 3.137e-04 0.2831783369
## 48 5.871e-05 0.66812502173   2.848e-04 0.43197677310 3.137e-04 0.7528169259
## 57 5.397e-04 0.13260147848   2.515e-04 0.52271460690 2.056e-03 0.2680365717
## 3  6.857e-05 0.93065927727   3.602e-04 0.39117665449 3.428e-04 0.9504295751
## 35 5.573e-05 0.99694607442   2.972e-04 0.59163370069 3.137e-04 0.9969460744
## 47 1.000e-04 0.35809201698   3.387e-04 0.66410956054 4.212e-04 0.5267002001
## 24 9.849e-01 0.00094181148   1.784e-03 0.21978715929 9.882e-01 0.0062787432
## 58 9.662e-04 0.16970528196   4.931e-04 1.00000000000 3.361e-03 0.3085550581
## 70 6.261e-04 0.44473041438   4.989e-04 1.00000000000 2.277e-03 0.6148194581
## 80 3.142e-03 0.03729817042   1.459e-03 0.42423330673 9.309e-03 0.1028915046
## 23 1.127e-02 0.15118839297   4.299e-03 0.19326976312 2.816e-02 0.2831783369
## 53 1.080e-03 0.07285056950   9.574e-04 1.00000000000 3.499e-03 0.1766074412
## 38 4.785e-04 0.19231155623   9.598e-04 1.00000000000 1.914e-03 0.3344548804
## 79 6.401e-01 0.00577434370   6.182e-03 0.36268282513 7.370e-01 0.0293679661
## 34 1.177e-03 0.45452960116   3.915e-03 0.60598316987 3.621e-03 0.6163113236
## 66 4.662e-03 0.00648692332   5.746e-03 0.46637586044 1.332e-02 0.0305266980
## 77 1.093e-03 0.62394087836   4.291e-03 0.68046728153 3.499e-03 0.7134487185
## 12 6.606e-02 0.01585742365   2.784e-02 0.12116483326 1.289e-01 0.0591286464
## 52 6.789e-02 0.10759431316   1.114e-02 0.30401560761 1.293e-01 0.2326363528
## 33 5.633e-03 0.28659474109   4.023e-03 1.00000000000 1.554e-02 0.4495603782
## 71 7.838e-03 0.84942276541   2.258e-02 0.19449513735 2.090e-02 0.8941292268
## 67 1.064e-01 0.02134489419   1.817e-02 0.36599826079 1.773e-01 0.0711496473
## 59 8.903e-01 0.01699948584   5.740e-02 0.20229595943 9.162e-01 0.0591286464
## 6  9.512e-03 0.14672804154   1.209e-02 1.00000000000 2.455e-02 0.2831783369
## 61 7.476e-01 0.01446874155   1.397e-02 1.00000000000 8.193e-01 0.0591286464
## 32 1.834e-01 0.00407688842   1.582e-02 1.00000000000 2.768e-01 0.0232965052
## 62 2.619e-02 0.39241154876   2.091e-02 1.00000000000 5.821e-02 0.5605879268
## 36 1.367e-02 0.02491547184   2.168e-02 1.00000000000 3.313e-02 0.0766629903
## 65 1.763e-02 0.44574410712   3.226e-02 1.00000000000 4.148e-02 0.6148194581
## 11 7.472e-01 0.01607830639   5.361e-02 1.00000000000 8.193e-01 0.0591286464
##    qallsubs_cv pglobal_cv qglobal_cv
## 39   0.000e+00  0.000e+00  0.000e+00
## 51   0.000e+00  0.000e+00  0.000e+00
## 76   0.000e+00  0.000e+00  0.000e+00
## 16   6.883e-12  1.022e-11  2.044e-10
## 1    1.036e-11  1.883e-11  3.012e-10
## 22   1.134e-04  3.012e-11  4.017e-10
## 9    9.803e-11  1.958e-10  2.238e-09
## 15   1.151e-07  3.065e-08  3.065e-07
## 75   1.106e-06  8.792e-07  7.815e-06
## 74   9.347e-06  1.860e-06  1.488e-05
## 50   8.366e-05  5.417e-06  3.940e-05
## 46   4.352e-06  7.603e-06  5.069e-05
## 21   8.072e-06  8.413e-06  5.177e-05
## 19   1.134e-04  3.107e-05  1.776e-04
## 69   1.251e-04  3.927e-05  2.094e-04
## 4    4.465e-05  4.520e-05  2.260e-04
## 7    2.453e-04  1.113e-04  5.239e-04
## 27   1.410e-04  1.222e-04  5.430e-04
## 41   7.090e-05  1.425e-04  6.001e-04
## 37   1.160e-04  2.002e-04  8.009e-04
## 14   1.840e-04  2.894e-04  1.063e-03
## 60   4.022e-04  2.924e-04  1.063e-03
## 72   3.551e-04  4.035e-04  1.403e-03
## 48   9.113e-04  1.231e-03  4.102e-03
## 57   8.384e-04  1.306e-03  4.180e-03
## 3    1.029e-03  1.390e-03  4.278e-03
## 35   9.144e-04  1.696e-03  5.025e-03
## 47   1.004e-03  2.114e-03  6.041e-03
## 24   4.198e-03  3.468e-03  9.566e-03
## 58   1.330e-03  4.248e-03  1.108e-02
## 70   1.330e-03  4.292e-03  1.108e-02
## 80   3.537e-03  5.191e-03  1.298e-02
## 23   9.050e-03  6.724e-03  1.630e-02
## 53   2.400e-03  7.613e-03  1.744e-02
## 38   2.400e-03  7.629e-03  1.744e-02
## 79   1.236e-02  1.592e-02  3.538e-02
## 34   8.940e-03  1.671e-02  3.613e-02
## 66   1.179e-02  1.855e-02  3.905e-02
## 77   9.050e-03  1.996e-02  4.094e-02
## 12   4.545e-02  2.257e-02  4.419e-02
## 52   2.173e-02  2.265e-02  4.419e-02
## 33   8.940e-03  2.621e-02  4.993e-02
## 71   3.763e-02  2.823e-02  5.252e-02
## 67   3.230e-02  3.998e-02  7.269e-02
## 59   8.831e-02  6.335e-02  1.102e-01
## 6    2.304e-02  6.549e-02  1.115e-01
## 61   2.599e-02  7.363e-02  1.227e-01
## 32   2.876e-02  8.140e-02  1.329e-01
## 62   3.637e-02  1.018e-01  1.573e-01
## 36   3.691e-02  1.048e-01  1.581e-01
## 65   5.162e-02  1.430e-01  2.037e-01
## 11   8.410e-02  2.105e-01  2.760e-01
```

```
# Function to calculate confidence intervals for dN/dS values per gene under the dNdScv model using profile likelihood.
 mm_comm_driver_dnds<- geneci(commpass_mm_rht, gene_list = drivers$Gene.Symbol, level = 0.95)
```

```
## Calculating CI95 across all genes...
```

```
 mm_comm_driver_dnds[mm_comm_driver_dnds$mis_low>1 | mm_comm_driver_dnds$tru_low>1,]
```

```
##        gene  mis_mle tru_mle  mis_low  tru_low mis_high tru_high
## 4     ARID2   0.2209   3.552  0.06857  1.52630   0.5131    6.870
## 7     BCL7A   1.3037   7.833  0.51043  2.91232   3.8566   23.792
## 9      BRAF   8.8874   0.000  5.00280  0.00000  11.1704    1.711
## 12   CDKN1B   2.6838  10.594  0.91367  1.76154   5.4383   32.712
## 14   CREBBP   0.3249   2.587  0.14848  1.11143   0.6048    5.002
## 15     CYLD   1.1680  11.454  0.59614  6.21983   2.0004   18.545
## 16     DIS3  10.5644   3.703  6.38122  1.32667  12.7380    7.959
## 19    DUSP2   1.0040   5.194  0.59544  2.58984   1.7363   10.088
## 21    EP300   0.2054   2.598  0.07366  1.11629   0.4415    5.024
## 22   FAM46C   5.8735   6.715  2.48994  1.34999  15.6169   28.798
## 23    FGFR3   2.9233   0.000  1.28491  0.00000   4.1954    1.865
## 24    FUBP1   0.9929   6.185  0.39108  2.43631   1.9202   11.962
## 27 HIST1H1E   6.4721   0.000  2.48528  0.00000  18.5349   12.152
## 32     IRF1   1.8395   7.197  0.67107  2.18127   3.7276   16.718
## 33     IRF4   4.2099   0.000  1.54526  0.00000   6.3338    3.383
## 36    KLHL6   3.3794   5.512  1.25203  1.25944   5.6838   14.941
## 37    KMT2B   0.3595   2.763  0.18016  1.26286   0.6306    5.144
## 39     KRAS 136.8413   0.000 40.61393  0.00000 154.0184    7.147
## 41      LTB   1.3956   7.851  0.72092  3.50906   2.8465   17.496
## 46      MAX  15.5495  23.652  6.14773  7.54738  22.0410   47.928
## 50   NFKBIA   1.5936  17.218  0.55034  6.76873   3.4251   33.299
## 51     NRAS 103.5569   0.000 31.51140  0.00000 117.9530    7.594
## 54     PIM1   3.0741   1.372  1.10054  0.06905   8.1723    9.377
## 55     POT1   2.0658   1.081  1.03625  0.06169   3.4009    4.761
## 56    PRDM1   1.7698   4.571  0.74237  1.05811   2.8224   11.853
## 57    PRKD2   3.0382   0.000  1.82168  0.00000   4.3065    1.698
## 58   PTPN11   3.4945   0.000  1.90406  0.00000   5.1648    2.037
## 59    RASA2   0.9528   3.655  0.43363  1.31059   1.7736    7.855
## 60      RB1   0.3359   4.947  0.08354  2.25070   0.8710    9.208
## 61    RFTN1   0.8413   4.452  0.30543  1.36602   2.4940   14.626
## 62    RPL10   4.8430   0.000  1.17356  0.00000   9.3031    5.505
## 65    RPS3A   3.3631   0.000  1.32293  0.00000   5.8987    6.607
## 66   SAMHD1   2.9039   4.721  1.53581  1.69054   4.4123   10.147
## 67    SETD2   0.6991   2.798  0.39771  1.19720   1.0737    5.412
## 69    SP140   1.2163   7.140  0.54830  3.27879   2.0831   11.975
## 70  TBC1D29   8.7967   0.000  2.20830  0.00000  18.8802    8.101
## 71    TCL1A   3.4667   1.240  1.35634  0.06408  11.1436    8.022
## 73     TGDS   2.5247   3.004  1.02369  0.49944   4.5512    9.275
## 74     TP53  10.6133   8.847  4.57393  2.71543  14.1544   19.015
## 75    TRAF2   1.1965  15.646  0.50952  7.53418   2.3140   27.441
## 76    TRAF3   6.4718  39.130  2.84101 16.86114   8.7207   55.149
## 79  ZFP36L1   0.6658  12.905  0.11704  2.26847   2.2848   44.286
## 80   ZNF292   0.4863   2.690  0.26749  1.06935   0.8005    5.452
```

```
  data("knownhotspots_hg19", package="dndscv") # Previously known hotspots
    known_hotspots = known_hotspots[sapply(known_hotspots, function(x) strsplit(x, split=":")[[1]][5]) %in% drivers$gene] # Known   hotspots in targetgenes
    hotspots_siteRHT_81_commpass = sitednds(commpass_mm, site_list = known_hotspots, min_recurr = 1, method = "LNP")
```

```
## [1] Site-wise overdispersed model accounting for trinucleotides and relative gene mutability...
```

```
##     Binning the rate vector: maximum deviation of 0.00071
```

```
## [2] Calculating site-wise dN/dS ratios and p-values...
```

```
##     Using the conservative bound of the confidence interval of the overdispersion parameter.
```

```
##     Peforming Restricted Hypothesis Testing on the input list of a-priori sites (numtests = length(site_list))
```

```
##     Mutations at known hotspots: 550 observed, 0.499 expected, obs/exp~1.1e+03 (CI95:1.01e+03,1.2e+03).
```

```
##     Modelling substitution rates using a Lognormal-Poisson: sig = 0.855 (upperbound = 0.874)
```

```
  hotspots_siteRHT_81_commpass
```

```
## $recursites
##    chr       pos ref mut   gene aachange           impact ref3_cod mut3_cod
## 1    1 115256529   T   C   NRAS     Q61R         Missense      CAA      CGA
## 2   12  25380275   T   G   KRAS     Q61H         Missense      AAG      ACG
## 3    7 140453136   A   T   BRAF    V600E         Missense      GTG      GAG
## 4    1 115258745   C   G   NRAS     G13R         Missense      TGG      TCG
## 5    1 115256530   G   T   NRAS     Q61K         Missense      ACA      AAA
## 6   12  25398284   C   T   KRAS     G12D         Missense      GGT      GAT
## 7   12  25398281   C   T   KRAS     G13D         Missense      GGC      GAC
## 8   12  25380276   T   A   KRAS     Q61L         Missense      CAA      CTA
## 9   12  25398285   C   G   KRAS     G12R         Missense      TGG      TCG
## 10  12  25398284   C   A   KRAS     G12V         Missense      GGT      GTT
## 11  12  25398284   C   G   KRAS     G12A         Missense      GGT      GCT
## 12  12  25380276   T   C   KRAS     Q61R         Missense      CAA      CGA
## 13   1 115256528   T   A   NRAS     Q61H         Missense      AAG      ATG
## 14   1 115256529   T   A   NRAS     Q61L         Missense      CAA      CTA
## 15  12  25380275   T   A   KRAS     Q61H         Missense      AAG      ATG
## 16   1 115256528   T   G   NRAS     Q61H         Missense      AAG      ACG
## 17  12  25398285   C   T   KRAS     G12S         Missense      TGG      TAG
## 18   1 115258744   C   T   NRAS     G13D         Missense      GGT      GAT
## 19   1 115258747   C   T   NRAS     G12D         Missense      GGT      GAT
## 20   6  37138423   G   C   PIM1     K24N         Missense      AGC      ACC
## 21  12  25398285   C   A   KRAS     G12C         Missense      TGG      TTG
## 22   7 140453155   C   T   BRAF    D594N         Missense      TGA      TAA
## 23   7 140453154   T   C   BRAF    D594G         Missense      GAT      GGT
## 24  12  25378561   G   A   KRAS    A146V         Missense      GCA      GTA
## 25   1 115258747   C   G   NRAS     G12A         Missense      GGT      GCT
## 26   1 115258748   C   T   NRAS     G12S         Missense      AGG      AAG
## 27  12  25378647   T   A   KRAS    K117N         Missense      AAT      ATT
## 28  12  25378562   C   T   KRAS    A146T         Missense      AGC      AAC
## 29   7 140481411   C   T   BRAF    G466E         Missense      GGA      GAA
## 30   4   1803564   C   T  FGFR3    R248C         Missense      GCG      GTG
## 31   2 209113113   G   A   IDH1    R132C         Missense      TCG      TTG
## 32  12  25378647   T   G   KRAS    K117N         Missense      AAT      ACT
## 33   7 140453134   T   C   BRAF    K601E         Missense      GAA      GGA
## 34  12  25398282   C   A   KRAS     G13C         Missense      TGG      TTG
## 35   4   1807889   A   G  FGFR3    K652E         Missense      GAA      GGA
## 36  17   7577538   C   T   TP53    R248Q         Missense      CGG      CAG
## 37   4   1806119   G   A  FGFR3    G382R         Missense      CGG      CAG
## 38   7 140481402   C   G   BRAF    G469A         Missense      GGA      GCA
## 39  17   7578253   C   A   TP53    G199V         Missense      GGA      GTA
## 40  17   7578449   C   T   TP53    A161T         Missense      GGC      GAC
## 41  12  25398262   C   G   KRAS     L19F         Missense      TGA      TCA
## 42  12 112888189   G   A PTPN11     E69K         Missense      GGA      GAA
## 43  17   7577545   T   G   TP53    M246L         Missense      CAT      CCT
## 44  17   7577574   T   G   TP53    Y236S         Missense      TAC      TCC
## 45  17   7577094   G   C   TP53    R282G         Missense      CCG      CGG
## 46  17   7578407   G   C   TP53    R175G         Missense      GCG      GGG
## 47   7 140453145   A   C   BRAF    L597R         Missense      CTA      CGA
## 48   1 115258748   C   A   NRAS     G12C         Missense      AGG      ATG
## 49  17   7578443   A   C   TP53    Y163D         Missense      CTA      CGA
## 50   2 198266834   T   C  SF3B1    K700E         Missense      GAA      GGA
## 51  17   7577129   A   G   TP53    F270S         Missense      TTT      TCT
## 52  12 112926888   G   T PTPN11    G503V         Missense      GGG      GTG
## 53  17   7574017   C   A   TP53    R337L         Missense      CGC      CTC
## 54  17   7578271   T   C   TP53    H193R         Missense      CAT      CGT
## 55   7 140481402   C   A   BRAF    G469V         Missense      GGA      GTA
## 56   7 140481411   C   A   BRAF    G466V         Missense      GGA      GTA
## 57  17   7578217   G   A   TP53    T211I         Missense      ACT      ATT
## 58  12  25380277   G   T   KRAS     Q61K         Missense      TCA      TAA
## 59   4   1803568   C   G  FGFR3    S249C         Missense      TCC      TGC
## 60  17   7578236   A   G   TP53    Y205H         Missense      GTA      GCA
## 61  17   7577580   T   C   TP53    Y234C         Missense      TAC      TGC
## 62  17   7578442   T   C   TP53    Y163C         Missense      TAC      TGC
## 63  17   7578553   T   C   TP53    Y126C         Missense      TAC      TGC
## 64  17   7577556   C   T   TP53    C242Y         Missense      TGC      TAC
## 65  17   7578526   C   T   TP53    C135Y         Missense      TGC      TAC
## 66  17   7578454   G   A   TP53    A159V         Missense      GCC      GTC
## 67  17   7577046   C   A   TP53    E298*         Nonsense      CGA      CTA
## 68  17   7577114   C   T   TP53    C275Y         Missense      TGT      TAT
## 69  17   7578203   C   T   TP53    V216M         Missense      TGT      TAT
## 70  17   7578370   C   T   TP53        . Essential_Splice      GGT      GAT
## 71  17   7574034   C   G   TP53        . Essential_Splice      AGA      ACA
## 72  17   7578534   C   G   TP53    K132N         Missense      AGA      ACA
## 73  17   7578550   G   A   TP53    S127F         Missense      TCC      TTC
## 74  17   7577085   C   T   TP53    E285K         Missense      AGA      AAA
## 75  17   7577097   C   T   TP53    D281N         Missense      AGA      AAA
## 76  17   7577099   C   T   TP53    R280K         Missense      AGA      AAA
## 77   3 178936091   G   A PIK3CA    E545K         Missense      TGA      TAA
## 78  17   7577094   G   A   TP53    R282W         Missense      CCG      CTG
## 79  17   7574018   G   A   TP53    R337C         Missense      GCG      GTG
## 80  17   7578406   C   T   TP53    R175H         Missense      CGC      CAC
## 81  17   7577139   G   A   TP53    R267W         Missense      ACG      ATG
## 82  17   7578212   G   A   TP53    R213*         Nonsense      TCG      TTG
##    freq        mu     dnds      pval      qval
## 1    69 0.0002926 235850.0 1.536e-47 9.398e-45
## 2    63 0.0003227 195212.5 4.193e-46 1.283e-43
## 3    31 0.0001686 183882.7 2.151e-44 4.389e-42
## 4    27 0.0001583 170606.3 1.807e-43 2.526e-41
## 5    52 0.0003802 136785.9 2.064e-43 2.526e-41
## 6    33 0.0006726  49065.4 4.456e-36 4.545e-34
## 7    30 0.0006428  46671.7 1.410e-35 1.232e-33
## 8    12 0.0001313  91363.1 1.877e-34 1.436e-32
## 9    12 0.0001576  76125.1 1.487e-33 1.011e-31
## 10   18 0.0004193  42925.2 1.871e-33 1.145e-31
## 11   12 0.0002357  50911.3 1.353e-31 7.530e-30
## 12   12 0.0002914  41179.7 1.413e-30 7.204e-29
## 13    9 0.0001796  50104.7 4.022e-28 1.894e-26
## 14    8 0.0001319  60668.6 4.163e-27 1.820e-25
## 15    8 0.0001789  44713.7 4.685e-26 1.911e-24
## 16    9 0.0003240  27777.6 7.340e-26 2.808e-24
## 17   10 0.0006234  16040.3 9.013e-25 3.245e-23
## 18    8 0.0006752  11847.8 1.628e-21 5.243e-20
## 19    8 0.0006752  11847.8 1.628e-21 5.243e-20
## 20    6 0.0002954  20310.5 8.487e-20 2.597e-18
## 21    5 0.0002750  18183.1 2.686e-17 7.828e-16
## 22    9 0.0038139   2359.8 7.307e-17 2.033e-15
## 23    5 0.0004052  12341.0 1.857e-16 4.940e-15
## 24    5 0.0006649   7520.0 2.188e-15 5.578e-14
## 25    4 0.0002366  16903.5 1.270e-14 3.109e-13
## 26    4 0.0006261   6389.2 6.187e-13 1.456e-11
## 27    3 0.0002504  11981.5 2.581e-11 5.850e-10
## 28    3 0.0006212   4829.4 3.930e-10 8.590e-09
## 29    3 0.0015542   1930.3 6.113e-09 1.290e-07
## 30    4 0.0065898    607.0 6.907e-09 1.409e-07
## 31    4 0.0071711    557.8 9.602e-09 1.896e-07
## 32    2 0.0001567  12763.8 2.633e-08 5.036e-07
## 33    2 0.0002582   7745.1 7.147e-08 1.326e-06
## 34    2 0.0002750   7273.3 8.107e-08 1.459e-06
## 35    2 0.0003165   6319.5 1.074e-07 1.878e-06
## 36    3 0.0043198    694.5 1.287e-07 2.187e-06
## 37    3 0.0047756    628.2 1.733e-07 2.866e-06
## 38    2 0.0004822   4147.7 2.491e-07 4.012e-06
## 39    2 0.0005978   3345.8 3.827e-07 6.006e-06
## 40    2 0.0007731   2586.8 6.398e-07 9.789e-06
## 41    2 0.0009168   2181.5 8.993e-07 1.342e-05
## 42    2 0.0014711   1359.5 2.312e-06 3.368e-05
## 43    1 0.0001481   6750.1 1.481e-04 2.107e-03
## 44    1 0.0001842   5428.5 1.842e-04 2.518e-03
## 45    1 0.0001852   5399.1 1.852e-04 2.518e-03
## 46    1 0.0002037   4908.0 2.037e-04 2.654e-03
## 47    1 0.0002039   4904.4 2.039e-04 2.654e-03
## 48    1 0.0002246   4452.6 2.245e-04 2.822e-03
## 49    1 0.0002260   4423.8 2.259e-04 2.822e-03
## 50    1 0.0002359   4238.7 2.358e-04 2.887e-03
## 51    1 0.0002656   3764.5 2.655e-04 3.133e-03
## 52    1 0.0002663   3754.5 2.662e-04 3.133e-03
## 53    1 0.0004273   2340.2 4.271e-04 4.932e-03
## 54    1 0.0005284   1892.5 5.281e-04 5.889e-03
## 55    1 0.0005392   1854.6 5.389e-04 5.889e-03
## 56    1 0.0005392   1854.6 5.389e-04 5.889e-03
## 57    1 0.0005539   1805.4 5.536e-04 5.944e-03
## 58    1 0.0005789   1727.4 5.785e-04 6.105e-03
## 59    1 0.0005893   1696.9 5.889e-04 6.109e-03
## 60    1 0.0006118   1634.5 6.114e-04 6.236e-03
## 61    1 0.0006846   1460.7 6.841e-04 6.625e-03
## 62    1 0.0006846   1460.7 6.841e-04 6.625e-03
## 63    1 0.0006846   1460.7 6.841e-04 6.625e-03
## 64    1 0.0007042   1420.1 7.037e-04 6.625e-03
## 65    1 0.0007042   1420.1 7.037e-04 6.625e-03
## 66    1 0.0007815   1279.6 7.808e-04 7.067e-03
## 67    1 0.0007918   1262.9 7.911e-04 7.067e-03
## 68    1 0.0008086   1236.7 8.079e-04 7.067e-03
## 69    1 0.0008086   1236.7 8.079e-04 7.067e-03
## 70    1 0.0008090   1236.1 8.083e-04 7.067e-03
## 71    1 0.0015353    651.3 1.533e-03 1.303e-02
## 72    1 0.0015353    651.3 1.533e-03 1.303e-02
## 73    1 0.0018163    550.6 1.813e-03 1.520e-02
## 74    1 0.0022391    446.6 2.234e-03 1.799e-02
## 75    1 0.0022391    446.6 2.234e-03 1.799e-02
## 76    1 0.0022391    446.6 2.234e-03 1.799e-02
## 77    1 0.0032967    303.3 3.285e-03 2.611e-02
## 78    1 0.0043777    228.4 4.357e-03 3.419e-02
## 79    1 0.0059609    167.8 5.923e-03 4.575e-02
## 80    1 0.0060190    166.1 5.980e-03 4.575e-02
## 81    1 0.0075058    133.2 7.446e-03 5.626e-02
## 82    1 0.0080987    123.5 8.029e-03 5.992e-02
## 
## $overdisp
##       MLE CI95_high 
##    0.8548    0.8738 
## 
## $fpr_nonsyn_q05
## NULL
## 
## $LL
## [1] -514106
## 
## $globaldnds_knownsites
##       obs       exp      dnds     cilow    cihigh 
##  550.0000    0.4994 1101.2407 1011.1174 1197.2422
```

# 5. Fisher's test frequency of patients with hotspots

```
# Let's recalculate for clarity:
 h_np = sitednds(d_NonPro, site_list = known_hotspots, min_recurr = 1, method = "LNP")
```

```
## [1] Site-wise overdispersed model accounting for trinucleotides and relative gene mutability...
```

```
##     Binning the rate vector: maximum deviation of 0.001
```

```
## [2] Calculating site-wise dN/dS ratios and p-values...
```

```
##     Using the conservative bound of the confidence interval of the overdispersion parameter.
```

```
##     Peforming Restricted Hypothesis Testing on the input list of a-priori sites (numtests = length(site_list))
```

```
##     Mutations at known hotspots: 2 observed, 0.00729 expected, obs/exp~274 (CI95:33.2,991).
```

```
##     Modelling substitution rates using a Lognormal-Poisson: sig = 0.01 (upperbound = 0.0963)
```

```
 h_pr = sitednds(d_Prog,   site_list = known_hotspots, min_recurr = 1, method = "LNP")
```

```
## [1] Site-wise overdispersed model accounting for trinucleotides and relative gene mutability...
```

```
##     Binning the rate vector: maximum deviation of 0.00108
```

```
## [2] Calculating site-wise dN/dS ratios and p-values...
```

```
##     Using the conservative bound of the confidence interval of the overdispersion parameter.
```

```
##     Peforming Restricted Hypothesis Testing on the input list of a-priori sites (numtests = length(site_list))
```

```
##     Mutations at known hotspots: 8 observed, 0.00765 expected, obs/exp~1.05e+03 (CI95:452,2.06e+03).
```

```
##     Modelling substitution rates using a Lognormal-Poisson: sig = 0.01 (upperbound = 0.099)
```

```
 h_mm = sitednds(d_mm,     site_list = known_hotspots, min_recurr = 1, method = "LNP")
```

```
## [1] Site-wise overdispersed model accounting for trinucleotides and relative gene mutability...
```

```
##     Binning the rate vector: maximum deviation of 0.00024
```

```
## [2] Calculating site-wise dN/dS ratios and p-values...
```

```
##     Using the conservative bound of the confidence interval of the overdispersion parameter.
```

```
##     Peforming Restricted Hypothesis Testing on the input list of a-priori sites (numtests = length(site_list))
```

```
##     Mutations at known hotspots: 27 observed, 0.025 expected, obs/exp~1.08e+03 (CI95:710,1.57e+03).
```

```
##     Modelling substitution rates using a Lognormal-Poisson: sig = 0.01 (upperbound = 0.0953)
```

```
 h_commpass = sitednds(commpass_mm,     site_list = known_hotspots, min_recurr = 1, method = "LNP")
```

```
## [1] Site-wise overdispersed model accounting for trinucleotides and relative gene mutability...
```

```
##     Binning the rate vector: maximum deviation of 0.00071
```

```
## [2] Calculating site-wise dN/dS ratios and p-values...
```

```
##     Using the conservative bound of the confidence interval of the overdispersion parameter.
```

```
##     Peforming Restricted Hypothesis Testing on the input list of a-priori sites (numtests = length(site_list))
```

```
##     Mutations at known hotspots: 550 observed, 0.499 expected, obs/exp~1.1e+03 (CI95:1.01e+03,1.2e+03).
```

```
##     Modelling substitution rates using a Lognormal-Poisson: sig = 0.855 (upperbound = 0.874)
```

```
# Creating some ids to cross-reference tables:
 h_np$recursites$id = paste(h_np$recursites$chr,h_np$recursites$pos,h_np$recursites$ref,h_np$recursites$mut,h_np$recursites$gene,sep=":")
 h_pr$recursites$id = paste(h_pr$recursites$chr,h_pr$recursites$pos,h_pr$recursites$ref,h_pr$recursites$mut,h_pr$recursites$gene,sep=":")
 h_mm$recursites$id = paste(h_mm$recursites$chr,h_mm$recursites$pos,h_mm$recursites$ref,h_mm$recursites$mut,h_mm$recursites$gene,sep=":")
 h_commpass$recursites$id = paste(h_commpass $recursites$chr,h_commpass $recursites$pos,h_commpass $recursites$ref,h_commpass $recursites$mut,h_commpass  $recursites$gene,sep=":")


 d_NonPro$annotmuts$id  = paste(d_NonPro$annotmuts$chr,d_NonPro$annotmuts$pos,d_NonPro$annotmuts$ref,d_NonPro$annotmuts$mut,d_NonPro$annotmuts$gene,sep=":")
 d_Prog$annotmuts$id    = paste(d_Prog$annotmuts$chr,d_Prog$annotmuts$pos,d_Prog$annotmuts$ref,d_Prog$annotmuts$mut,d_Prog$annotmuts$gene,sep=":")
 d_mm$annotmuts$id      = paste(d_mm$annotmuts$chr,d_mm$annotmuts$pos,d_mm$annotmuts$ref,d_mm$annotmuts$mut,d_mm$annotmuts$gene,sep=":")
 commpass_mm$annotmuts$id  = paste(commpass_mm$annotmuts$chr,commpass_mm$annotmuts$pos,commpass_mm$annotmuts$ref,commpass_mm$annotmuts$mut,commpass_mm$annotmuts$gene,sep=":")


# Number of patients in each group. Modify accordingly if needed
 num_patients_np = length(unique(muts_NonPro$sample))
 num_patients_pr = length(unique(muts_progressed$sample))
 num_patients_mm = length(unique(muts_mm$sample))
 num_patients_commpass = length(unique(commpass2$sample))

# Number of patients with hotspots in each group:
 num_patients_np_with_hotspots = length(unique(d_NonPro$annotmuts[which(d_NonPro$annotmuts$id%in%h_np$recursites$id),"sampleID"]))
 num_patients_pr_with_hotspots = length(unique(d_Prog$annotmuts[which(d_Prog$annotmuts$id%in%h_pr$recursites$id),"sampleID"]))
 num_patients_mm_with_hotspots = length(unique(d_mm$annotmuts[which(d_mm$annotmuts$id%in%h_mm$recursites$id),"sampleID"]))
 num_patients_commpass_mm_with_hotspots = length(unique(commpass_mm$annotmuts[which(commpass_mm$annotmuts$id%in%h_commpass$recursites$id),"sampleID"]))

# Fisher's tests:

mat = matrix(nrow=2,ncol=2)
    # NonProg vs Prog
    mat[1,] = c(num_patients_np_with_hotspots,num_patients_np-num_patients_np_with_hotspots)
    mat[2,] = c(num_patients_pr_with_hotspots,num_patients_pr-num_patients_pr_with_hotspots)
    fisher.test(mat)
```

```
## 
##  Fisher's Exact Test for Count Data
## 
## data:  mat
## p-value = 0.0005
## alternative hypothesis: true odds ratio is not equal to 1
## 95 percent confidence interval:
##  0.006043 0.392828
## sample estimates:
## odds ratio 
##    0.06617
```

```
    # p-value = 0.0004612445

    # NonProg vs MM
    mat[1,] = c(num_patients_np_with_hotspots,num_patients_np-num_patients_np_with_hotspots)
    mat[2,] = c(num_patients_mm_with_hotspots,num_patients_mm-num_patients_mm_with_hotspots)
    fisher.test(mat)
```

```
## 
##  Fisher's Exact Test for Count Data
## 
## data:  mat
## p-value = 0.0001
## alternative hypothesis: true odds ratio is not equal to 1
## 95 percent confidence interval:
##  0.009894 0.394142
## sample estimates:
## odds ratio 
##    0.09056
```

```
    # p-value = 0.000116883
    
    # Prog vs MM
    mat[1,] = c(num_patients_pr_with_hotspots,num_patients_pr-num_patients_pr_with_hotspots)
    mat[2,] = c(num_patients_mm_with_hotspots,num_patients_mm-num_patients_mm_with_hotspots)
    fisher.test(mat)
```

```
## 
##  Fisher's Exact Test for Count Data
## 
## data:  mat
## p-value = 0.6
## alternative hypothesis: true odds ratio is not equal to 1
## 95 percent confidence interval:
##  0.4408 4.4268
## sample estimates:
## odds ratio 
##      1.422
```

```
    # p-value = 0.5950621
    
    
    # NonProg vs commpass
    mat[1,] = c(num_patients_np_with_hotspots,num_patients_pr-num_patients_np_with_hotspots)
    mat[2,] = c(num_patients_commpass_mm_with_hotspots,num_patients_commpass-num_patients_commpass_mm_with_hotspots)
    fisher.test(mat)
```

```
## 
##  Fisher's Exact Test for Count Data
## 
## data:  mat
## p-value = 0.0008
## alternative hypothesis: true odds ratio is not equal to 1
## 95 percent confidence interval:
##  0.01376 0.52353
## sample estimates:
## odds ratio 
##     0.1232
```

```
    # p-value = 0.0007528831
    
    
    # Prog vs commpass
    mat[1,] = c(num_patients_pr_with_hotspots,num_patients_pr-num_patients_pr_with_hotspots)
    mat[2,] = c(num_patients_commpass_mm_with_hotspots,num_patients_commpass-num_patients_commpass_mm_with_hotspots)
    fisher.test(mat)
```

```
## 
##  Fisher's Exact Test for Count Data
## 
## data:  mat
## p-value = 0.6
## alternative hypothesis: true odds ratio is not equal to 1
## 95 percent confidence interval:
##  0.263 2.097
## sample estimates:
## odds ratio 
##     0.7605
```

```
    # p-value = 0.6462823
    
    # MM vs commpass
    mat[1,] = c(num_patients_mm_with_hotspots,num_patients_mm-num_patients_mm_with_hotspots)
    mat[2,] = c(num_patients_commpass_mm_with_hotspots,num_patients_commpass-num_patients_commpass_mm_with_hotspots)
    fisher.test(mat)
```

```
## 
##  Fisher's Exact Test for Count Data
## 
## data:  mat
## p-value = 0.01
## alternative hypothesis: true odds ratio is not equal to 1
## 95 percent confidence interval:
##  0.3165 0.8794
## sample estimates:
## odds ratio 
##     0.5329
```

```
    # p-value = 0.01015666
    
    
    par(mfrow=c(1,1), mar=c(10,8,5,10), xpd=T)
    x<- barplot(cbind( c(num_patients_np_with_hotspots,num_patients_np-num_patients_np_with_hotspots),
                   c(num_patients_pr_with_hotspots,num_patients_pr-num_patients_pr_with_hotspots),
                   c(num_patients_mm_with_hotspots,num_patients_mm-num_patients_mm_with_hotspots),
                   c(num_patients_commpass_mm_with_hotspots,num_patients_commpass-num_patients_commpass_mm_with_hotspots)),
            , beside=TRUE, las=2, cex.axis=1.5, col=c("coral4","darkolivegreen4"))
    
    text(x=(x[1,]+x[2,])/2, y=-10, labels = c("MGUS-SMM SD", "MGUS-SMM PD", "MM WGS", "MM WXS"), col= c("dodgerblue", "darkorchid", "brown4","brown4"),
         adj=1, srt=60, cex=1.5)
    mtext(side=2, text="Number of patients", line=4, cex=1.5)
    legend("topright", legend=c("hotspot", "non-hotspot"), col=c("coral4","darkolivegreen4"), 
           pch=15, inset=c(-0.3,0.0), bty =   "n", border = "n", cex=1.5)
```
